# Usability evaluation of an innovative device for hypodermoclysis in a simulated scenario with nurses^*^

**DOI:** 10.1590/1518-8345.8289.4930

**Published:** 2026-10-09

**Authors:** Thayna de Assis Barros S. Thiago, Eduardo José Ferreira Santos, Pedro Miguel dos Santos Dinis Parreira, Anabela Salgueiro-Oliveira, Juliana Faria Campos, Inês Franco de Almeida, Rafael Alves Bernardes, Marcelle Miranda da Silva

**Affiliations:** 1 Universidade Federal do Rio de Janeiro, Escola de Enfermagem Anna Nery, Rio de Janeiro, RJ, Brazil.; 2 Scholarship holder at the Coordenação de Aperfeiçoamento de Pessoal de Nível Superior (CAPES), Brazil.; 3 Instituto Politécnico de Viseu, Escola de Saúde, Viseu, Portugal.; 4 Escola Superior de Enfermagem de Coimbra, Unidade de Investigação em Ciências da Saúde, Enfermagem, Coimbra, Portugal.; 5 Scholarship holder at the Escola Superior de Enfermagem de Coimbra, Unidade de Investigação em Ciências da Saúde, Enfermagem, Portugal.; 6 Universidade Católica Portuguesa, Centro de Investigação Interdisciplinar em Saúde, Lisboa, Portugal.

**Keywords:** User-centered Design, Subcutaneous Infusions, Equipment and Supplies, Hypodermoclysis, Technology, Inventions.

## Abstract

**Objective:**

to evaluate the usability and operational performance of the Subcutaneous Hydration Device prototype in a simulated scenario with nurses, as well as to gather input for its continuous refinement.

**Method:**

pre-experimental, qualitative, and quantitative usability study using a simulated scenario, semi-structured interview, and focus group. Conducted in a laboratory with nine participants with experience in subcutaneous therapy. A checklist was used as a guide for conducting the simulated scenario and evaluating the operational performance of the prototype, as well as a script for the interview and focus group. Thematic content analysis was applied.

**Results:**

varied perceptions of the prototype were revealed, identifying strengths and weaknesses. Most participants demonstrated satisfaction with the ease of handling and expressed confidence in its possible implementation in clinical practice. Improvements related to ergonomics and functionality were suggested, such as the inclusion of accessories to reinforce stability, in addition to identifying the home environment and adults/elderly people as possible contexts and beneficiaries of the Subcutaneous Hydration Device.

**Conclusion:**

the study showed satisfactory performance of the Subcutaneous Hydration Device prototype in a simulated scenario and generated positive satisfaction among nurses. The potential of the Subcutaneous Hydration Device to improve nursing care, especially at home, was highlighted.

## Highlights

**(1)** The SHD prototype demonstrated effectiveness in simulations with experienced nurses.

**(2)** Nurses reported high satisfaction with the ease of use of the SHD prototype.

**(3)** The SHD has potential for home care, favoring dehospitalization.

**(4)** Improvements in ergonomics and functionality were suggested to enhance the SHD.

## Introduction

Dehydration is a common fluid disorder that affects individuals across a variety of settings, from the home to institutional environments^1^. Linked to increased morbidity and mortality, especially in vulnerable groups such as children and the elderly, its relevance and the need for a careful approach are highlighted^1^ ^-^ ^2^.

Although adequate water intake is crucial to reduce the risk of dehydration, in cases where oral rehydration is insufficient, intravenous or subcutaneous fluid administration becomes essential^2^. Hypodermoclysis, particularly, emerges as a valuable option for patients with mild to moderate dehydration, especially those with venous access difficulties or restrictions on oral administration and absorption^3^.

Although hypodermoclysis offers advantages, its application remains limited and underutilized due to practical challenges, particularly in the home environment^2^ ^-^ ^3^. The Subcutaneous Hydration Device (SHD) emerges as an innovative solution to improve the clinical application of the hypodermoclysis technique with safety and practicality. Conceived by undergraduates from a nursing institution in central Portugal, as part of the innovation module of the Professional Life Integration course, the SHD was registered with the National Institute of Industrial Property (INPI, Portuguese acronym) as a utility model, underscoring its relevance to contemporary clinical practice^4^.

The SHD fits the definition of a medical device intended for use in the prevention, treatment, or relief of a specific medical condition, in this context, mild to moderate dehydration, characterized by a loss of up to 10% of fluids^5^. Thus, in accordance with the legal requirements established by the Portuguese National Authority for Medicines and Health Products (INFARMED) and European guidelines, the SHD has been classified as a Class III device, specifically as a short-term invasive device^6^.

In the context of innovation, a prototype is a physical or conceptual representation of a design idea that goes through iterative stages of testing and refinement to meet user expectations. It is essential to test prototypes to improve their effectiveness and usability, especially in medical devices^7^ ^-^ ^8^. The SHD prototype was developed using commercially available technologies, in accordance with the specifications of the SHD utility model (Figure 1), and underwent an initial focus group phase with healthcare professionals to optimize its design and assess its potential market acceptance^9^.

According to the SHD utility model, the device consists of a closed system prefilled with 0.9% saline solution (SS0.9%), and is ready for use and single use. However, due to the improvised nature of the prototyping process, some extra components, such as a clamp and the second lumen (4 and 9) were retained, but were not considered in this study.

For assembly, a 400 mL elastomeric infusion pump was chosen, with a predefined infusion flow rate of 10 mL/h (1). The equipment includes a reservoir inside (2), the administration set (3) with a clamp (4), a particle and air filter (5), and two lumens for connection (9). The access device contains a catheter (6), flexible and ergonomic wings (7), and a safety device (8) for removing the needle (Figure 1).

**Figure 1:**
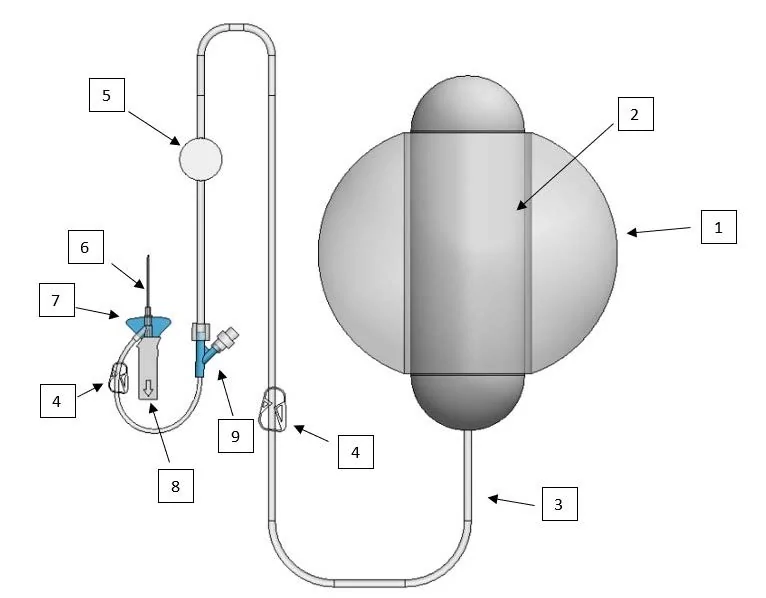
Illustration of the semi-functional prototype of the Subcutaneous Hydration Device

To advance the SHD prototyping process, this study aims to evaluate the usability and operational performance of the SHD prototype in a simulated scenario with nurses, and to gather input for its continued refinement.

## Method

### Study design

A pre-experimental study was conducted to evaluate the usability of the SHD, using a combination of qualitative and quantitative approaches, in accordance with the Methodological Guidelines for Medical Device Evaluation Studies^10^. Focusing on the operational domain, we analyzed user-device interaction, developing parameters to measure satisfaction and operational performance.

We conducted a single-group experiment to evaluate the usability of the SHD prototype, focusing on the subcutaneous administration of SS0.9% through clinical simulation, followed by semi-structured interviews. Participants were subsequently invited to join a focus group to further refine recommendations for improving the SHD prototype. To ensure methodological quality, we followed the Strengthening the Reporting of Observational Studies in Epidemiology (STROBE) guidelines adapted for simulation studies, according to the research design^11^.

### Study location

The first phase of this study took place in the skills laboratory of the nursing program at a federal university in Rio de Janeiro, Brazil. In this environment, we conducted both a prototype evaluation in a simulated scenario and semi-structured interviews. The focus group, in turn, was held in the computer lab of a graduate nursing program in the same city. Both labs were reserved exclusively for study activities, ensuring an environment free from noise and external interruptions.

### Sample definition

Participants were recruited via snowball sampling, beginning with nurses affiliated with the research group, to which the principal investigator belonged, and then expanding through successive referrals to other potential participants. This process occurred in two waves of referrals, until the new recommendations began to produce similar profiles, without adding relevant characteristics to the phenomenon under study^12^.

In this process, 26 nurses were referred, but only 16 were eligible, and seven were unable to attend the data collection site. Thus, the first phase of the study included nine participants. Although there is no established consensus on the ideal sample size that guarantees, by itself, a fully reliable usability assessment^13^, this number was determined by the criterion of thematic saturation of the data and the homogeneity of the sample (nurses experienced in hypodermoclysis). After the data collection was completed, the participants were invited to join the focus group.

During the recruitment process, we reviewed the curricula vitae of referred nurses and asked about their practical experience with the hypodermoclysis technique. Invitations were sent by email, and after acceptance, participants chose the most convenient time for in-person data collection.

### Selection criteria

The target population consisted of nurses with experience in caring for patients undergoing subcutaneous infusions, such as hypodermoclysis. Nurses with self-reported clinical experience in hypodermoclysis were eligible, regardless of their area of practice, while those who had previous contact with the product to be tested were excluded.

### Data collection

Simulated scenario, semi-structured interview, and focus group

Data collection was conducted between March and April 2023, preceded by a pilot test in September 2022, in a laboratory at a private hospital in Rio de Janeiro. This test involved the participation of a nurse experienced in hypodermoclysis and a nursing professor specializing in clinical simulation.

Initially, two testing stages were planned, but the pilot test demonstrated that the first stage -hypodermoclysis using the conventional method with an electronic infusion pump- was unnecessary. In addition, the test highlighted the need for a manual connection between the devices, to enable reuse of the elastomeric infusion pump, because permanent bonding of the administration set to the single-use needle precluded reuse. Thus, testing in a simulated scenario focused exclusively on evaluating the usability of the SHD prototype.

The data collection process involved four stages: (1) reading and signing the Informed Consent Form, along with the Image and Voice Release Form; (2) characterization of the participant’s professional profile through a self-administered questionnaire with nine open and closed questions; (3) testing the prototype in a simulated clinical setting; and (4) semi-structured interviews.

On the scheduled day and time, participants attended the laboratory individually, where they received an introduction to the research and the SHD. They signed the Consent Forms and completed the questionnaire related to their professional profile.

In the simulated environment, participants received instructions for the proposed activity, guided by the simulated scenario guide, which simulated the care of a moderate-complexity patient in a medical ward. The researcher allowed participants time to handle the SHD, clarify any doubts, and receive additional instructions before proceeding to the subsequent steps.

After the scenario introduction, participants simulated the application of hypodermoclysis for an SS0.9% infusion on a simulation manikin, following the clinical case instructions. The simulated environment was designed to replicate a realistic clinical scenario, incorporating technological resources such as hypodermoclysis materials and other items representative of a medical clinic hospital environment.

The simulator selected was a unisex manikin, designed for nursing education, capable of simulating a wide range of basic procedures, including dressings, catheterization, bed bathing, and subcutaneous drug administration. For the SHD prototype test scenarios, the materials shown in Figure 2 were used.

**Figure 2:** Materials for testing in a simulated scenario. Rio de Janeiro, RJ, Brazil, 2023

Items	Amount
Easypump^®^ II LT* elastomeric infusion pumps from B|Braun with 400 ml and 10 ml/h infusion flow rate, containing SS0.9%	2
BD Nexiva™ Closed IV Catheter System, double-lumen, closed-system intravenous catheters	9
Vitamedical^®^ waterproof transparent film dressing roll	1
Micropore^TM^ surgical tape	1
Pairs of procedural gloves	9
Packages of gauze pad containing 10 units each	9
Bottle of 70% alcohol solution	1
Pens	2
Sheets with the answer form	-
Mobile phone used as a stopwatch	1

*LT = Long-Term

During testing, participants were observed by the principal investigator, who used a checklist based on the Standard Operating Procedure (SOP) for hypodermoclysis from of a university hospital in Rio de Janeiro, which underwent a pilot test to ensure alignment with nursing practice. This checklist verified the mandatory steps for subcutaneous installation with the SHD: handwashing, use of gloves, skin antisepsis, skinfold creation, catheter insertion, opening of clamps, and securing the device with sterile film.

The task was considered successfully completed when all mandatory steps were performed, and the participant verbally declared the end of the procedure. In addition to recording errors/successes and the time spent in the simulation, the checklist evaluated the operational performance of the prototype, as detailed in the results section (Table 2).

After completing the testing, participants underwent a semi-structured interview to assess their satisfaction with the SHD prototype and share additional insights and contributions. Thus, satisfaction was measured based on the positive and negative impressions expressed during the interviews, which were audio-recorded and transcribed verbatim, thus concluding the data collection process for this session. 

The focus group took place on April 19, 2023, with the principal investigator as moderator, a master’s student as an observer, and six previously interviewed participants. Of the nine guests, three declined due to scheduling conflicts, without compromising to thematic diversity, as the topics discussed had already emerged in the individual interviews.

A detailed, semi-structured guide was prepared to guide the focus group, outlining the steps to be followed. Initially, participants were gathered, and the researcher introduced the session proposal and format. At the start, participants had the opportunity to introduce themselves and interact before the activity began.

Then, the researcher led the discussion according to the planned questions, ensuring everyone’s participation and allowing time for additional questions. The questions addressed four thematic areas: general characteristics of the SHD; components of the SHD; context of SHD use; and nursing management and administration. The focus group lasted one hour and twenty minutes, ending when all questions had been addressed. The complete guide is available as supplementary material.

### Data processing and analysis

The audio recordings of the semi-structured interviews and focus group were transcribed in full and analyzed jointly, as they presented complementary content. The quantitative data from the questionnaires and the checklist were analyzed using the Statistical Program for the Social Sciences (SPSS) software, version 22.0, employing descriptive analysis.

The interviews and focus group were transcribed in full by the principal investigator and sent to the participants for validation, with no changes requested by them. The qualitative data were analyzed using Qualitative Data Analysis Software (webQDA^®^) by the research group, following the thematic content analysis method proposed by Bardin: pre-analysis, exploration of the material, treatment of the results, inference, and interpretation^14^. The results were organized into thematic units defined *a priori*, based on the study objectives, which were: satisfaction, safety, application context/target audience, and prototype components.

### Ethical considerations

The project was approved by the Research Ethics Committee of the Anna Nery School of Nursing/São Francisco de Assis Health Care Institute/Federal University of Rio de Janeiro (CEP-EEAN/HESFA/UFRJ) (Approval No. 5,786,249), the certificate of which is attached to this submission. All participants were kept anonymous and identified by specific codes: “N” for nurses, followed by a number, in the semi-structured interviews; and “FGP” for focus group participants, followed by a number. This measure was taken to preserve participants’ integrity and privacy.

## Results

Regarding the nine participants’ professional profiles, shown in Table 1, the majority (66.7%) were female, with an average age of 33 years, ranging from 27-64 years. Five had a master’s degree (55.6%) and one had a doctorate (11.1%). Most (66.7%) had more than six years of nursing experience. Five had one to five years of experience in hypodermoclysis, and only one had more than ten years. Areas of experience included oncology (the most common, with five participants), palliative care, emergency care, and gerontology.

The simulation activity, observed with the aid of the checklist (Table 2) and timed, revealed a mean execution time of 5 minutes and 42 seconds among participants. The maximum time recorded for completion was 10 minutes and 2 seconds, while the minimum was 3 minutes and 24 seconds, as illustrated in Figure 3. All participants successfully completed the simulation, although two did not verify whether the clamps were closed.

**Table 1:** Sociodemographic and professional data of the participants. Rio de Janeiro, RJ, Brazil, 2023

Variables	Frequency (n)	Percentage (%)
Age (years)		
27-44	6	66.7
45-64	3	33.3

Gender		
Female	6	66.7
Male	3	33.3

Highest academic degree		
Specialization	3	33.3
Master’s degree	5	55.6
Doctorate	1	11.1

Years of experience as a nurse		
2 to 5 years	3	33.3
6 to 10 years	2	22.2
More than 10 years	4	44.4

Years of experience with hypodermoclysis		
Less than 1 year	2	22.2
1 to 5 years	5	55.6
6 to 10 years	1	11.1
More than 10 years	1	11.1

Received training in hypodermoclysis		
Yes	8	88.9
No	1	11.1

Self-rated knowledge of hypodermoclysis		
Excellent	1	11.1
Very good	2	22.2
Good	6	66.7

**Table 2:** Checklist of procedures performed during the simulated scenario testing phase. Rio de Janeiro, RJ, Brazil, 2023

Procedures	Frequency (n)	Percentage (%)
Wash your hands with soap and water or apply 70% alcohol solution	9	100
Put on the procedural gloves	9	100
Open the package containing the prototype of the Subcutaneous Hydration Device	9	100
Ensure that the clamps are closed		
Yes	7	77.8
No	2	22.2
Perform skin antisepsis with gauze soaked in 2% aqueous chlorhexidine.	9	100
Pinch a skin fold for subcutaneous access.	9	100
Insert the over-the-needle catheter at an angle of 30º to 45º with the bevel facing up	9	100
Open the administration set clamps and release the flow.	9	100
Secure the device with sterile transparent film.	9	100
Task completed.	9	100

**Figure 3:**
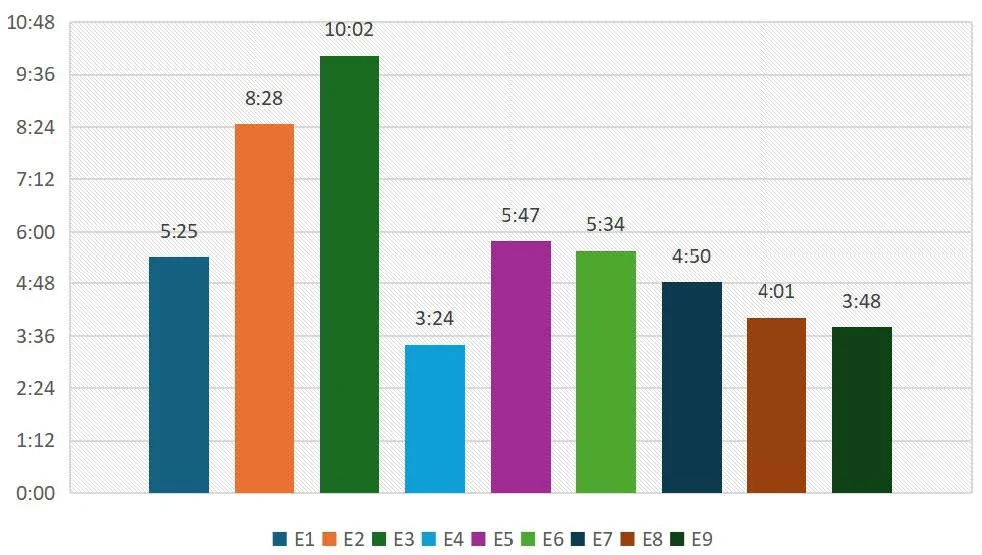
Procedure execution time by nurses during the simulation

Participants expressed satisfaction with the device’s ease of handling, noting that, even without prior training, they encountered no difficulties during the simulation.

*I thought it was great, compact, nice. I think it’s great to handle, very good, I have no difficulty handling it. (N5)*

*I found it very interesting and, especially, easy to use, intuitive, and, most importantly, it does not allow you to manipulate it and, above all, change any pre-set, pre-use settings. (N8)*

The innovation incorporating the elastomeric infusion pump was considered the central feature of the SHD prototype. In addition to enhancing system compactness and simplifying material preparation and use, this innovation replaces complex, bulky equipment, such as electronic infusion pumps.

*Certainly, the first point is that the infusion pump is a cumbersome device that you must move around, and it is difficult to use, as is the gravity administration set, which many people do not understand how it works and end up lowering it, which is not effective. This pump is easier, you just put it in and that’s it. (N2)*

*Something I think is super important: we eliminate the use of the electronic infusion pump, which is a rigid technology, expensive, difficult to use and handle, as well as being difficult to transport, and which makes an unbearable noise and disturbs the patient. (FGP3)*

When asked about their intention to use the device, participants expressed confidence that the SHD prototype would contribute significantly to their professional activities when integrated into the work environment, indicating their satisfaction and enthusiasm for its implementation.

*So, for me, I would be very happy to use it, not to mention leaving the device installed and everything else. For stable patients, it’s great. (N5)*

*I think it will be very useful. I would use it, yes. (N6)*

*It’s perfect! For home use? Perfect. For home caregivers? I think it’s perfect. (N7)*

The prototype received unanimous praise from all participants. Its ability to ensure a contamination-free environment, thanks to its closed-system design, as well as its ease of handling for both professionals and caregivers, was highlighted.

*I believe that the level of infection is very low. If I were the head of the ward, I would be very happy [...] I found it interesting that there is no direct contact with the needle. If there is infection, it will probably be due to the person who performed the technique [...] so it would be less of a problem for the quality nurse. (N5)*

*In terms of process, I think it ends up having the same process as other equipment. But in relation to the patient, because it is a closed system, you don’t have to manipulate it, you don’t have to change the drip rate, the infusion time [...]. I think it is safer for the patient. (N9)*

*What favors the safety of the device is, for example, that it is a closed system and you cannot change the ml/h. At home, they can open it accidentally and cause subcutaneous edema. Safety is: regardless of the height of the device, it will work because it works under pressure, and regardless of the movement made, the ml/h will be guaranteed. (FGP6)*

The SHD proposal as a ready-to-use device was recognized as a significant advantage, as it simplifies healthcare professionals’ tasks while promoting patient safety.

*I think it’s very positive and interesting, mainly because it doesn’t control the ml/h and comes pre-made. We already know for sure what will be infused. (N8)*

*I think it makes the team’s work easier, it comes filled, and it’s faster. (FGP2)*

To keep this system inviolable and mitigate the risk of misuse, participants identified weaknesses in the prototype that could be addressed to ensure the proposed safety.

*I found the elastomeric pump inlet very easy to open. This will pass through other hands [...] there is a risk of contaminating the system during filling and breaking the sterility of the device if this continues, it must be kept closed. (FGP3)*

*There will be people at home who will take a syringe and try to inject something here. (FGP5)*

*I think the top of the pump could follow the same pattern as the bottom. Have this part here where you put the saline solution and then put a closed cap on it so that no one can put anything in. (FGP4).*

The participants in the study showed interest in implementing the SHD in their workplaces. More specifically, professionals working in home care services, covering both oncological and geriatric palliative care, highlighted the potential relevance of SHD in this context. Above all, they emphasized that the device would be well accepted in home care, where patients receive care from family members or informal caregivers.

*I think it will be a very beneficial device, especially for home use and private care, or perhaps even for a long-term care facility [...]. Now, speaking from a gerontology point of view, to avoid hospitalization, sometimes you just want to see if the patient’s problem is dehydration [...] I think it’s perfect. (N6)*

*At home, I think it’s perfect, for safety, for ease of handling, even aesthetically pleasant. At home, they don’t have IV support, so they have to improvise to use gravity-fed systems. [...] So, if I have to hydrate this patient at home, they will usually stay in bed. If they need to go to the bathroom or do something, they will be able to do so easily with this device. For home use, I think the device is perfect. (N7)*

SHD was considered a facilitator of the transition of patient care from the hospital sector to home care. In addition, it was considered a potential strategy to prevent hospitalization among patients receiving home care.

*I think it facilitates the discharge of patients who are in the hospital just for hydration. (FGP2)*

*What we are talking about is a specific issue or, perhaps, transitional care. The patient is hospitalized, they are going to be discharged, and you have to train the family on how to use the device. So, you put it on once and train them, show the family how to do it, how it will be done at home. (FGP6)*

*It prevents the hospitalization of those who are at home and dehydrated. You put the device on and hydrate them. You hydrate the patient and, by treating dehydration, help improve venous visibility, and the process of caring for the patient is facilitated by this device. (FGP3)*

To standardize the characteristics of the prototype components, they identified the need to define an initial target audience for the SHD. They considered it interesting to prioritize adult patients because their needs are less specific than those of other age groups.

*I think the adults are better, the pediatrics has too much variation, you have 1-year-olds and 10-year-olds, and the infusion volume is different for each. (FGP4)*

*I think that, at first, the ideal would be the adult audience, because it is in the initial phase of the device’s development, creating many variations at the moment would not be good for the market. (FGP5)*

The pump’s weight, combined with its rounded design and the lack of an accessory to secure the prototype, was identified as a set of factors that increases the risk of the device falling, thereby compromising patient safety.

*What worries me a little is the weight of the pump, [...] any movement that may occur, perhaps due to its weight at the beginning of the infusion, may cause the administration set to retract. (N2)*

*When performing the technique, my biggest difficulty was figuring out where to place the pump, because if I leave it here on the bed, the patient will move in bed, knock it over, and that force could break the device. (N3)*

*I think that without the support, it will roll and fall. When you put it on the table, I got nervous, because I think it will roll and fall. (FGP2)*

To resolve this issue and minimize the risk of the SHD falling, participants proposed including complementary accessories in the kit.

*If it had one, it would also be a plus for the device: a small bag, something like that. For caregivers, whether professional or family caregivers, this would help 100%, in addition to trying to explore the patient’s independence as much as possible. (N2)*

*Put a hook on it if you have to put it on the bed, shorten the strap if the patient needs to walk, put a longer strap on it. (FGP2)*

*The ideal would be a strap that could be adjusted in size and a bag as an accessory that could be purchased separately. (FGP6)*

The elastomeric infusion pump, initially perceived as having insufficient volume, was proposed to be offered in multiple volumes to meet diverse patient needs and avoid both product and financial waste.

*If we standardize it to a single volume, we would be wasting saline solution and unnecessarily incurring high costs; the ideal would be to have different volumes to allow for choice (...) I think there should be a product with several presentations. (FGP6)*

*Today we have several IV bags with different volumes, so why not standardize the traditional volumes? 100, 200, and 500? If the prescribed volume is 200 and the bag has 500, there will be a need to monitor it to stop the flow at the right time, which would be more work. (FGP3)*

Furthermore, given the perception that the prototype’s infusion time is long-40 hours-it was recommended that subcutaneous hydration protocols be followed, such as administering 500 mL of 0.9% saline solution over 24 hours.

*I found the infusion time to be very long [...] the device must have a certain degree of control so that you can also reverse the patient’s dehydration in a timely manner [...] I see the infusion time of this device as a problem. (FGP3)*

*I think up to 80 ml/h would be comfortable, 120 is not feasible (...) normally, we infuse 500 ml in 24 hours, if you want more volume, we put another bag in another site. But it is standardized for 24 hours. (FGP5)*

All participants praised the intravenous catheter selected for the prototype for its practicality and safety, despite the subcutaneous site. There was consensus to standardize the SHD to the smallest available gauge, rejecting 90º needles due to difficulty in maintaining the device subcutaneous placement during infusion.

*When we work with the subcutaneous site under gravity, the smallest gauge does not work, and we have to resort to larger ones. As this device is pressure-driven, I believe that it is possible to reduce the gauge, which will cause less pain at the time of puncture and will be suitable for all patients. (FGP6)*

*I think that if it were 90°, if you don’t make a proper fold, you could end up going through the subcutaneous tissue. (N2)*

*I don’t see a problem with installation; I believe it would be similar to insulin. I see a problem with the maintenance of this device because, even if the insulin injection misses the site, it will be a very small volume, unlike a 500 ml volume. (FGP2)*

Recognizing the need to simplify the procedure for users without technical experience, such as informal caregivers responsible for home hypodermoclysis in palliative care programs, participants proposed the optional inclusion of a puncture guide in the SHD kit. The aim was, above all, to assist in achieving the correct angle of 30º or 45º during insertion, considered the most critical point of the procedure.

*I think it could even come inside the device kit. I imagine it as a piece to guide the puncture. One side is 15°, another 30°, and the other 45°, and the informal caregiver will follow the instructions on which degree to use. (FGP5)*

The final prototype presentation has two clamps due to the manual joining of commercially available materials. Although unintentional, this duplication sparked debate among participants. After considering the safety aspects of the clamp, they reached a consensus that the best option was to position the clamp close to the elastomeric infusion pump and change its color to improve visibility.

*I would prioritize keeping it close to the pump, which I think exposes the patient less. (N6)*

*The patient could even accidentally activate the clamp, leaving it open close to the patient, who could change body position and close the clamp. (FGP4)*

*True, create a strategy to make the clamp more visible to caregivers. (FGP4)*

## Discussion

The SHD study yielded relevant conclusions regarding the prototype’s performance in a simulated scenario and the feasibility of this innovation in clinical practice. The results showed a positive reception by nursing professionals, highlighting its ease of use, safety, and potential to optimize the quality of care. Satisfaction with the handling of the prototype and the perception of safety associated with the closed system indicate that the SHD can offer an effective and convenient alternative for fluid administration in patients with mild to moderate dehydration.

Considering the centrality of the user in the health innovation process, participants’ suggestions for improving the SHD include: adding accessories to reinforce the stability of the device and making different volumes of SS0.9% available, which could be customized in the future according to the patient and their needs, after the completion of clinical studies. Despite the emphasis on the ease of use of the SHD, the lack of support for securing the elastomeric infusion pump was identified as a weakness, indicating a potential risk of complications, such as accidental displacement or disconnection.

Although the current infusion rate of the prototype is within the recommended limits^15^, participants suggested a flow rate of 20 mL/h, as they considered it more effective for managing patient hydration. Regarding catheter dwell time, although it is described as short in the literature^2^, the risk analysis showed that this duration is adequate to minimize complications at the puncture site. Therefore, standardizing the SHD to 24 hours, as suggested by the participants, would enhance the device’s clinical and operational safety.

A key point identified in the study, which has significant implications for healthcare professionals, was the definition of the context of use and the target population benefiting from the SHD: the home environment and adult patients. Participants viewed the SHD as a viable alternative to facilitate home care, aligning with safe dehospitalization. Prolonged hospital stays increase patients’ risks, including the development of hospital-acquired conditions, underscoring dehospitalization as an important goal for healthcare teams and the healthcare system itself^16^ ^-^ ^17^.

In this scenario, the SHD was perceived as a technology that facilitates continuity of home care, particularly in programs in which family caregivers perform hypodermoclysis under supervision and technical guidance. It is essential to recognize the fundamental role of caregivers, often family members, who require technical support and health education to ensure adequate home care^18^, including subcutaneous hydration. Participants considered the SHD a viable proposal for informal caregivers, due to its simplicity and features that can facilitate safe management^19^ ^-^ ^20^.

This exploratory study, which is pioneering in the development and evaluation of the SHD, revealed positive perceptions among participants regarding its potential to facilitate dehospitalization and prevent avoidable hospitalizations resulting from dehydration. Given that hospitalization is associated with functional and locomotor capacity loss in the elderly person, the relevance of strategies that enable home care is reinforced^21^.

For this potential to be fully realized, it will be necessary to integrate knowledge between the health and industrial sectors in the process of developing and improving the device, as in the development of technologies such as hospital pajamas, which also relied on an interdisciplinary team for its design and implementation^22^.

This study has some methodological limitations. The sample was restricted to graduate-level nurses, which limited the diversity of profiles and specialties represented. Professionals working in pediatrics, intensive care, and the operating room, for example, were excluded, thereby reducing the scope of the assessment of the SHD’s applicability across clinical settings. In addition, end users, patients, and caregivers were not included in the usability assessment process, which is an important gap to be overcome in future studies.

Despite these limitations, the results of this study offer relevant input for advancing scientific knowledge in health and nursing by examining the collaborative development of a technology for subcutaneous fluid administration for rehydration. The SHD has demonstrated the potential to expand and strengthen home care, enhance patient and caregiver autonomy, and reduce the burden on health services, particularly in the context of an aging population and chronic diseases.

## Conclusion

Usability evaluation is crucial for identifying potential problems that users of medical devices may encounter. This study demonstrated positive operational performance of the SHD prototype in a simulated scenario, generating satisfaction among participating nurses. Although some design concerns were highlighted, participants showed interest in implementing the SHD in their work environments. Thus, the results of this study pave the way for future research on the SHD and highlight its potential to improve nursing care, particularly for adults and elderly people, and to expand home care.

## Data Availability

All data generated or analysed during this study are included in this published article.
