# Supplementary material for: Usability evaluation of an innovative device for hypodermoclysis in a simulated scenario with nurses
Source: Rev Lat Am Enfermagem. 2026 Oct 9;34:e4930. doi: 10.1590/1518-8345.8289.4930 (PMC13652531; doi:10.1590/1518-8345.8289.4930)
Supplement: Translation

# Translation: Avaliação de usabilidade de um dispositivo inovador para hipodermóclise em cenário simulado com enfermeiros^*^

**DOI:** 10.1590/1518-8345.8289.4931

**Authors:** Thayna de Assis Barros S. Thiago, Eduardo José Ferreira Santos, Pedro Miguel dos Santos Dinis Parreira, Anabela Salgueiro-Oliveira, Juliana Faria Campos, Inês Franco de Almeida, Rafael Alves Bernardes, Marcelle Miranda da Silva

**Affiliations:** 1 Universidade Federal do Rio de Janeiro, Escola de Enfermagem Anna Nery, Rio de Janeiro, RJ, Brasil.; 2 Bolsista da Coordenação de Aperfeiçoamento de Pessoal de Nível Superior (CAPES), Brasil.; 3 Instituto Politécnico de Viseu, Escola de Saúde, Viseu, Portugal.; 4 Escola Superior de Enfermagem de Coimbra, Unidade de Investigação em Ciências da Saúde, Enfermagem, Coimbra, Portugal.; 5 Bolsista da Escola Superior de Enfermagem de Coimbra, Unidade de Investigação em Ciências da Saúde, Enfermagem, Portugal.; 6 Universidade Católica Portuguesa, Centro de Investigação Interdisciplinar em Saúde, Lisboa, Portugal.

**Keywords:** Design Centrado no Usuário, Infusões Subcutâneas, Equipamentos e Provisões, Hipodermóclise, Tecnologia, Invenções

## Abstract

**Objetivo:**

avaliar a usabilidade e o desempenho operacional do protótipo do Dispositivo de Hidratação Subcutânea em cenário simulado com enfermeiros, bem como reunir subsídios para seu contínuo refinamento.

**Método:**

estudo de usabilidade pré-experimental, qualitativo e quantitativo, utilizando cenário simulado, entrevista semiestruturada e grupo focal. Realizado em laboratório, com nove participantes com experiência em terapia subcutânea. Um *checklist* foi utilizado como guia para a condução do cenário simulado e para a avaliação do desempenho operacional do protótipo, bem como um roteiro para a entrevista e o grupo focal. Aplicou-se a análise de conteúdo na modalidade temática.

**Resultados:**

revelaram-se percepções variadas sobre o protótipo, identificando forças e fragilidades. A maioria demonstrou satisfação com a facilidade de manipulação e expressou confiança em sua possível implementação na prática clínica. Foram sugeridas melhorias relacionadas à ergonomia e funcionalidade, como a inclusão de acessórios para reforçar a estabilidade, além de identificarem o ambiente domiciliar e adultos/idosos como possíveis contextos e beneficiários do Dispositivo de Hidratação Subcutânea.

**Conclusão:**

o estudo evidenciou desempenho satisfatório do protótipo do Dispositivo de Hidratação Subcutânea em cenário simulado e gerou satisfação positiva entre os enfermeiros. Destacou-se o potencial do Dispositivo de Hidratação Subcutânea para aprimorar os cuidados de enfermagem, especialmente em domicílio.

## Destaques

**(1)** O protótipo do DHS demonstrou eficácia na simulação com enfermeiros experientes.

**(2)** Enfermeiros relataram alta satisfação com a facilidade de uso do protótipo do DHS.

**(3)** O DHS possui potencial para cuidados domiciliares, favorecendo a desospitalização.

**(4)** Foram sugeridas melhorias na ergonomia e funcionalidade para aprimorar o DHS.

## Introdução

A desidratação é um transtorno hídrico frequente que impacta indivíduos em uma variedade de cenários, desde o ambiente domiciliar até o institucional^1^. Vinculada ao aumento da morbidade e da mortalidade, sobretudo em grupos vulneráveis como crianças e idosos, destaca sua relevância e a necessidade de uma abordagem cuidadosa^1^ ^-^ ^2^.

Embora a ingestão adequada de água seja crucial para reduzir o risco de desidratação, em casos em que a reidratação oral não é suficiente, a administração de fluidos por via intravenosa ou subcutânea se torna essencial^2^. A hipodermóclise, particularmente, emerge como uma opção valiosa para pacientes com desidratação leve a moderada, especialmente àqueles com dificuldades de acesso venoso ou restrições à administração e à absorção oral^3^.

Mesmo que a hipodermóclise ofereça vantagens, sua aplicação ainda é limitada e subutilizada devido a desafios práticos, especialmente em ambiente domiciliar^2^ ^-^ ^3^. O Dispositivo de Hidratação Subcutânea (DHS) surge como uma solução inovadora para aprimorar a aplicação clínica da técnica da hipodermóclise com segurança e praticidade. Idealizado por estudantes de graduação de uma instituição de enfermagem da região Centro de Portugal, como parte do módulo de inovação da unidade curricular de Integração à Vida Profissional, o DHS obteve registro no Instituto Nacional da Propriedade Industrial (INPI) como modelo de utilidade, destacando sua relevância para a prática clínica contemporânea^4^.

O DHS se enquadra na definição de dispositivo médico destinado ao uso na prevenção, tratamento ou alívio de uma condição médica específica, neste contexto, a desidratação leve a moderada, caracterizada por perda de até 10% de fluidos^5^. Dessa forma, seguindo os requisitos legais estabelecidos pela Autoridade Nacional do Medicamento e Produtos de Saúde (INFARMED) de Portugal e as diretrizes europeias, o DHS foi categorizado como um dispositivo de Classe III, especificamente classificado como dispositivo invasivo de curta duração^6^.

No contexto da inovação, um protótipo é uma representação física ou conceitual de uma ideia de design, que passa por etapas iterativas de teste e refinamento para atender às expectativas do usuário. É essencial testar protótipos para aprimorar sua eficácia e usabilidade, principalmente em dispositivos médicos^7^ ^-^ ^8^. O protótipo do DHS foi desenvolvido com base em tecnologias disponíveis no mercado, seguindo as especificações do modelo de utilidade do DHS (Figura 1), e foi submetido a uma fase inicial de grupo focal com profissionais de saúde para otimizar seu design e avaliar sua aceitação potencial no mercado^9^.

Conforme o modelo de utilidade do DHS, o dispositivo é composto por um sistema fechado e pré-preenchido com soro fisiológico a 0,9% (SF0,9%), pronto para uso e de utilização única. Entretanto, devido à natureza improvisada do processo de prototipagem, alguns componentes extras, como um *clamp* e o segundo lúmen (4 e 9) foram mantidos, mas não foram considerados na realização deste estudo. 

Para a montagem, optou-se por uma bomba de infusão elastomérica de 400 mL, com o fluxo pré-definido de 10 mL/h (1). O equipamento inclui um reservatório interno (2), um equipo (3) com *clamp* (4), filtro de partículas e ar (5) e dois lúmens para conexão (9). O dispositivo de acesso apresenta um cateter (6), asas flexíveis e ergonômicas (7) e um dispositivo de segurança (8) para retirada da agulha (Figura 1).

**Figura 1:** Ilustração do protótipo semifuncional do Dispositivo de Hidratação Subcutânea

Para dar continuidade ao processo de prototipagem do DHS, este estudo tem como objetivos avaliar a usabilidade e o desempenho operacional do protótipo do DHS em cenário simulado com enfermeiros, bem como reunir subsídios para seu contínuo refinamento.

## Método

### Delineamento do estudo

Um estudo pré-experimental foi conduzido para avaliar a usabilidade do DHS, combinando abordagens qualitativas e quantitativas, conforme as Diretrizes Metodológicas para Estudos de Avaliação de Equipamentos Médico-assistenciais^10^. Com foco no domínio operacional, analisamos a interação usuário-dispositivo, desenvolvendo parâmetros para medir a satisfação e o desempenho operacional.

Conduzimos um experimento em grupo único para avaliar a usabilidade do protótipo do DHS, com foco na administração subcutânea de SF0,9%, por meio de simulação clínica, seguida por entrevistas semiestruturadas. Os participantes foram posteriormente convidados a participar de um grupo focal, a fim de aprofundar as recomendações de melhoria do protótipo do DHS. Para garantir a qualidade metodológica, seguimos as diretrizes da extensão *Strengthening the Reporting of Observational Studies in Epidemiology* (STROBE) adaptadas para estudos com simulação, conforme o delineamento da pesquisa^11^.

### Local do estudo

A primeira fase deste estudo ocorreu no laboratório de atividades práticas do curso de enfermagem de uma universidade federal no Rio de Janeiro, Brasil. Neste ambiente, conduzimos tanto a avaliação do protótipo em um cenário simulado quanto as entrevistas semiestruturadas. Por sua vez, o grupo focal foi realizado no laboratório de informática de um programa de pós-graduação em enfermagem na mesma cidade. Ambos os laboratórios foram reservados exclusivamente para as atividades do estudo, garantindo um ambiente livre de ruídos e de interrupções externas.

### Definição da amostra

Os participantes foram recrutados pela técnica *snowball*, iniciando-se com enfermeiros membros do grupo de pesquisa ao qual a pesquisadora principal estava associada, e a partir deles, expandindo-se progressivamente por meio de indicações sucessivas de outros potenciais participantes. Esse processo ocorreu em duas ondas de indicação, até que as novas recomendações passaram a repetir perfis semelhantes, sem acrescentar características relevantes ao fenômeno estudado^12^. 

Neste percurso, foram indicados 26 enfermeiros, mas apenas 16 eram elegíveis e sete não possuíam disponibilidade para comparecer ao local de coleta de dados. Assim, a primeira fase do estudo contou com nove participantes. Embora não haja consenso estabelecido sobre o tamanho da amostra ideal que garanta, por si só, uma avaliação de usabilidade totalmente confiável^13^, esse número foi determinado pelo critério de saturação temática dos dados e pela homogeneidade da amostra (enfermeiros experientes em hipodermóclise). Após o encerramento da coleta de dados, os participantes foram convidados a integrar o grupo focal. 

Durante o processo de recrutamento, consultamos os currículos dos enfermeiros indicados e os questionamos sobre sua experiência prática na técnica da hipodermóclise. Os convites foram enviados por e-mail e, após a aceitação, os participantes escolheram o momento mais adequado para a coleta de dados presencialmente.

### Critérios de seleção

A população-alvo consistiu em enfermeiros com experiência em atendimento a pacientes submetidos a infusões subcutâneas, como a hipodermóclise. Foram elegíveis enfermeiros com experiência clínica em hipodermóclise - autorrelatada - independentemente de sua área de atuação, sendo excluídos aqueles que tiveram contato anterior com o produto a ser testado. 

### Coleta de dados

Cenário simulado, entrevista semiestruturada e grupo focal

A coleta de dados foi realizada entre março e abril de 2023, precedida de um teste-piloto realizado em setembro de 2022, em um laboratório de um hospital privado no Rio de Janeiro. Esse teste contou com a participação de uma enfermeira experiente em hipodermóclise e de uma docente de enfermagem especializada em simulação clínica. 

Inicialmente, estavam previstas duas etapas de testagem, porém o teste-piloto demonstrou que a primeira etapa - que envolvia a realização da hipodermóclise pelo método convencional, com bomba de perfusão elétrica - era desnecessária. Além disso, o teste evidenciou a necessidade de conexão manual entre os dispositivos, para permitir o reaproveitamento da bomba de infusão elastomérica, uma vez que a colagem definitiva do circuito à agulha de uso único inviabilizava o reuso. Dessa forma, a testagem em cenário simulado concentrou-se exclusivamente na avaliação de usabilidade do protótipo do DHS.

O processo de coleta de dados envolveu quatro etapas: (1) a leitura e assinatura do Termo de Consentimento Livre e Esclarecido, juntamente com o Termo de Consentimento para Uso de Imagem e Voz; (2) a caracterização do perfil profissional do participante por meio de um questionário autoaplicável com nove questões abertas e fechadas; (3) a testagem do protótipo em um cenário clínico simulado; e (4) entrevistas semiestruturadas.

No dia e horário agendados, os participantes compareceram ao laboratório individualmente, onde receberam introdução sobre a pesquisa e sobre o DHS. Assinaram os Termos de Consentimento e preencheram o questionário relacionado ao seu perfil profissional.

No ambiente simulado, os participantes receberam instruções sobre a atividade proposta, guiadas pelo roteiro do cenário simulado, que consistia em simular o cuidado a um paciente de média complexidade em uma enfermaria de clínica médica. A pesquisadora disponibilizou um momento para que os participantes pudessem manipular o DHS, esclarecer dúvidas e receber instruções adicionais antes das etapas subsequentes.

Após a introdução ao cenário, os participantes simularam a aplicação da hipodermóclise para infusão de SF0,9% em um manequim simulador, conforme as instruções do caso clínico. O ambiente simulado foi projetado para replicar um cenário clínico realista, incorporando recursos tecnológicos, como materiais para hipodermóclise e outros representativos do ambiente hospitalar de uma clínica médica.

O simulador selecionado foi um manequim bissexual, designado para educação em enfermagem, capaz de simular uma ampla gama de procedimentos básicos, incluindo curativos, passagem de sondas, banho no leito e administração de medicamentos subcutâneos. Para os cenários de teste do protótipo do DHS, foram utilizados os materiais dispostos na Figura 2.

**Figura 2:** Materiais para a testagem em cenário simulado. Rio de Janeiro, RJ, Brasil, 2023

Itens	Quantidade
Bombas de infusão elastomérica Easypump^®^ II LT* da B|Braun com 400 ml e 10 ml/h de fluxo de infusão, contendo SF0,9%	2
Cateteres endovenosos de duplo lúmen, de sistema fechado BD Nexiva™ Closed IV Catheter System	9
Rolo de curativo de filme transparente impermeável Vitamedical^®^	1
Fita de curativo Micropore^MR^	1
Pares de luvas de procedimentos	9
Pacotes de compressas de gazes contendo 10 unidades cada	9
Frasco de álcool 70%	1
Canetas	2
Folhas com o formulário de respostas	-
Dispositivo de telefonia móvel para utilização de cronômetro	1

*LT = *Long-Term*

Durante a testagem, os participantes foram observados pela pesquisadora principal, que utilizou um *checklist* fundamentado no Procedimento Operacional Padrão (POP) de um hospital universitário do Rio de Janeiro para hipodermóclise e que foi submetido a um teste-piloto para garantir o alinhamento com a prática de enfermagem. Este *checklist* verificou as etapas obrigatórias da instalação subcutânea com o DHS: lavagem das mãos, uso de luvas, antissepsia da pele, realização da prega, inserção do cateter, abertura dos *clamps* e fixação do dispositivo com filme estéril. 

A tarefa foi considerada concluída com sucesso quando todas as etapas obrigatórias foram executadas e o participante declarou verbalmente o término do procedimento. Além de registrar erros/acertos e o tempo despendido na simulação, o *checklist* avaliou o desempenho operacional do protótipo, conforme detalhado na seção dos resultados (Tabela 2). 

Após a finalização da testagem, os participantes passaram por uma entrevista semiestruturada para avaliar sua satisfação em relação ao uso do protótipo do DHS e compartilhar percepções e contribuições adicionais. Desse modo, a satisfação foi mensurada a partir das impressões positivas e negativas expressas durante as entrevistas, que foram gravadas em áudio e transcritas na íntegra, encerrando, assim, o processo de coleta de dados neste encontro. 

O grupo focal ocorreu no dia 19 de abril de 2023, com a pesquisadora principal como moderadora, uma estudante de mestrado como observadora e seis participantes previamente entrevistados. Dos nove convidados, três declinaram por incompatibilidade de agenda, sem prejuízo à diversidade temática, pois os eixos discutidos já haviam emergido nas entrevistas individuais.

Para a condução do grupo focal, elaborou-se um roteiro semiestruturado detalhado, apresentando as etapas a serem seguidas. Inicialmente, os participantes foram reunidos, e a pesquisadora introduziu a proposta e o formato da sessão. Durante este momento inicial, os participantes tiveram a oportunidade de se apresentar e interagir, antes do início efetivo da atividade. 

Em seguida, a pesquisadora liderou a discussão conforme as perguntas planejadas, assegurando a participação de todos e abrindo espaço para questionamentos adicionais. As perguntas abordaram quatro eixos temáticos: características gerais do DHS; componentes do DHS; contexto de uso do DHS e gestão e gerenciamento de enfermagem. O grupo focal teve a duração de uma hora e vinte minutos, encerrando-se quando todas as questões foram esgotadas. O roteiro completo está disponibilizado como material suplementar.

### Tratamento e análise de dados

Os áudios das entrevistas semiestruturadas e do grupo focal foram transcritos na íntegra e analisados em conjunto, por apresentarem conteúdos complementares. Os dados quantitativos dos questionários e do *checklist* foram analisados utilizando o *software Statistical Program for the Social Sciences* (SPSS) versão 22.0, por meio de análise descritiva. 

As entrevistas e o grupo focal foram transcritos integralmente pela pesquisadora principal e enviados aos participantes para validação, não havendo alterações solicitadas por eles. A análise dos dados qualitativos foi realizada utilizando o *Qualitative Data Analysis Software* (webQDA^®^) pelo grupo de pesquisadores, seguindo o método de análise de conteúdo temática proposto por Bardin: pré-análise, exploração do material e tratamento dos resultados obtidos, inferência e interpretação^14^. Os resultados foram organizados em unidades temáticas definidas *a priori*, com base nos objetivos do estudo, sendo elas: satisfação, segurança, contexto de aplicação/público-alvo e componentes do protótipo.

### Considerações éticas

O projeto recebeu aprovação do Comitê de Ética em Pesquisa da Escola de Enfermagem Anna Nery/Instituto de Atenção à Saúde São Francisco de Assis/Universidade Federal do Rio de Janeiro (CEP-EEAN/HESFA/UFRJ) (Parecer N.º 5.786.249), cujo parecer está anexado à presente submissão. Todos os participantes foram mantidos anônimos, sendo identificados por códigos específicos: “E”, para enfermeiros, seguido de um número, nas entrevistas semiestruturadas; e “PG”, para participantes do grupo focal, seguido de um número. Essa medida foi tomada para preservar a integridade e privacidade dos participantes.

## Resultados

Acerca do perfil profissional dos nove participantes, exposto na Tabela 1, a maioria (66,7%) era do sexo feminino, com idade média de 33 anos, variando de 27 a 64 anos. Cinco possuíam mestrado (55,6%) e um doutorado (11,1%). A maioria (66,7%) tinha mais de seis anos de experiência como enfermeiros. Cinco tinham de um a cinco anos de experiência em hipodermóclise, e somente um tinha mais de dez anos. As áreas de experiência incluíam oncologia (a mais comum, com cinco participantes), cuidados paliativos, emergência e gerontologia.

A atividade de simulação, observada com o auxílio do *checklist* (Tabela 2) e cronometrada, revelou um tempo médio de execução de 5 minutos e 42 segundos entre os participantes. O tempo máximo registrado para a conclusão foi de 10 minutos e 2 segundos, enquanto o mínimo foi de 3 minutos e 24 segundos, conforme ilustrado na Figura 3. Todos os participantes concluíram a simulação com sucesso, embora dois não tenham verificado se os *clamps* estavam fechados.

**Tabela 1:** Dados sociodemográficos e profissionais dos participantes. Rio de Janeiro, RJ, Brasil, 2023

Variáveis	Frequência (n)	Porcentagem (%)
Idade (anos)		
27-44	6	66.7
45-64	3	33.3

Sexo		
Feminino	6	66.7
Masculino	3	33.3

Maior titulação acadêmica		
Especialização	3	33.3
Mestrado	5	55.6
Doutorado	1	11.1

Tempo exercendo o cargo de enfermeiro		
2 to 5 anos	3	33.3
6 to 10 anos	2	22.2
Mais de 10 anos	4	44.4

Quanto tempo possui de experiência com hipodermóclise		
Menos de 1 ano	2	22.2
1 to 5 anos	5	55.6
6 to 10 anos	1	11.1
Mais de 10 anos	1	11.1

Recebeu algum treinamento de hipodermóclise		
Sim	8	88.9
Não	1	11.1

Qual nível de conhecimento você considera ter em relação a hipodermóclise?		
Excelente	1	11.1
Muito bom	2	22.2
Bom	6	66.7

**Tabela 2:** *Checklist* dos procedimentos realizados na etapa de teste em cenário simulado. Rio de Janeiro, RJ, Brasil, 2023

Procedimentos	Frequência (n)	Porcentagem (%)
Lavar as mãos com água e sabão ou aplicar álcool a 70% nas mãos	9	100
Calçar as luvas de procedimento	9	100
Abrir o pacote do protótipo do Dispositivo de Hidratação Subcutânea	9	100
Certificar-se de que os *clamps* estão fechados		
Sim	7	77.8
Não	2	22.2
Realizar antissepsia da pele com gaze embebida em clorexidina aquosa 2%	9	100
Tracionar uma prega de pele para acesso subcutâneo	9	100
Introduzir o cateter não agulhado em um ângulo de 30º a 45º com o bisel para cima	9	100
Abrir os clamps da linha de extensão e liberar o fluxo	9	100
Fixar o dispositivo com filme transparente estéril	9	100
Completou a tarefa	9	100

**Figura 3:** Tempo de execução do procedimento-teste pelos enfermeiros na simulação

Os participantes expressaram satisfação com a facilidade de manipulação do dispositivo, observando que, mesmo sem receberem treinamento prévio para sua operação, não enfrentaram dificuldades durante a simulação.

*Achei-a ótima, compacta, achei-a bonitinha. Acho que ela é ótima para manusear, muito boa, não tenho dificuldade nenhuma de manuseio. (E5)*

*Achei muito interessante e, principalmente, fácil de utilizar, intuitivo e, principalmente, não dá margem para que você esteja manipulando-o e, principalmente, alterando qualquer objetivo pré-fabricado, de pré-utilização. (E8)*

A inovação que incorpora a bomba de infusão elastomérica foi considerada a característica central do protótipo do DHS. Além de contribuir para a composição de um sistema compacto e de simplificar a etapa de preparação/utilização dos materiais, essa inovação substitui equipamentos complexos e volumosos, como a bomba de perfusão elétrica.

*Com certeza, o primeiro ponto é que a bomba infusora é um trambolho, que você tem que estar deslocando, e é difícil de mexer, assim como o circuito por gravidade, que muitos não entendem como funciona e acabam abaixando-o, e isso não é efetivo. Essa bomba é mais fácil, é só colocar e pronto. (E2)*

*Algo que eu acho super importante: a gente elimina o uso da bomba infusora, que é uma tecnologia dura, cara, difícil de ser utilizada e manuseada, além de ser difícil de transportar, e que faz um barulho insuportável e perturba o paciente. (PG3)*

Ao serem questionados sobre a intenção de uso, os participantes expressaram confiança de que o protótipo do DHS contribuiria significativamente para suas atividades profissionais ao ser integrado ao ambiente de trabalho, evidenciando sua satisfação e entusiasmo com a perspectiva de sua implementação.

*Então, para mim, eu ficaria muito feliz em usar, sem contar aquela coisa de deixar o dispositivo instalado e tudo mais, para o paciente estável, ele é ótimo. (E5)*

*Eu acho que vai ser muito útil, eu usaria, sim. (E6)*

*É perfeito! Para o domicílio? Perfeito. Para o cuidador domiciliar? Eu acho que é perfeito. (E7)*

O protótipo recebeu elogios unânimes por parte de todos os participantes. Destacaram-se sua capacidade de garantir um ambiente livre de contaminação, graças ao seu design como sistema fechado, bem como a facilidade de manuseio, tanto para profissionais quanto para cuidadores.

*Creio que o nível de infecção seja muito baixo, se eu fosse chefe da enfermaria, ficaria muito feliz [...] eu achei interessante o fato de não ter contato direto com a agulha. Se for por infecção, provavelmente será por quem fez a técnica [...] então seria menos um problema para o enfermeiro da qualidade. (E5)*

*Em termos de processo, eu acho que acaba tendo o mesmo processo do que os outros equipamentos. Mas em relação ao paciente, por ser sistema fechado, de não ter que manipular, de não mudar a questão do gotejamento, do tempo de infusão [...]. Eu acho que é mais seguro para o paciente. (E9)*

*O que favorece a segurança do dispositivo é, por exemplo, ele ser sistema fechado e você não poder alterar o ml/h. No domicílio, eles podem abrir sem querer e gerar aquele edema no subcutâneo. A segurança está: independente da altura do dispositivo, ele vai funcionar, porque ele funciona sob pressão, e independente do movimento que se faça, o ml/h vai estar garantido. (PG6)*

A proposta do DHS como dispositivo pronto para uso foi reconhecida como uma vantagem significativa, por simplificar as tarefas dos profissionais de saúde enquanto favorece a segurança do paciente.

*Acho que muito positivo e interessante, principalmente por não controlar o ml/h e já vir pré-fabricado. A gente já tem a certeza do que vai ser infundido. (E8)*

*Acho que ele facilita o trabalho da equipe, ele vir preenchido, e é mais rápido. (PG2)*

Para manter esse sistema inviolável e mitigar as possibilidades de mau uso do dispositivo, os participantes identificaram pontos de fragilidade no protótipo que poderiam ser alterados para garantir a segurança proposta.

*Eu achei a porta de entrada da bomba elastomérica muito fácil de abrir. Isso aqui vai passar por outras mãos [...] há o risco de contaminar o sistema durante o preenchimento e de romper a esterilidade do dispositivo se isso continuar assim, tem que deixar fechado. (PG3)*

*Vai ter gente, no domicílio, que vai pegar uma seringa e tentar injetar alguma coisa aqui. (PG5)*

*Eu acho que a parte de cima da bomba poderia seguir o mesmo padrão da parte de baixo. Ter essa parte aqui em que se coloca o soro e, depois, colocar uma tampa fechada, para que ninguém coloque nada. (PG4).*

Os participantes do estudo demonstraram interesse na implementação do DHS em seus locais de trabalho. Mais especificamente, os profissionais que atuam na prestação de serviços de assistência domiciliar, abrangendo tanto cuidados paliativos oncológicos quanto geriátricos, destacaram a potencial relevância do DHS nesse contexto. Enfatizaram, sobretudo, que o dispositivo teria uma boa aceitação para a assistência domiciliar, onde os pacientes recebem cuidados por membros da família ou cuidadores informais.

*Eu acho que ele vai ser um dispositivo muito benéfico, principalmente para uso domiciliar e em atendimentos particulares, ou talvez até para uma instituição de longa permanência [...]. Agora, falando do ponto de vista da gerontologia, para você evitar uma internação, você às vezes só quer ver se o problema daquele paciente é uma desidratação [...] eu acho perfeito. (E6)*

*No domicílio, eu acho perfeito, pela segurança, pela praticidade de manipular, até a questão estética ele é agradável. No domicílio eles não têm suporte de soro, ficam adaptando para fazer por gravidade. [...] Então, se eu tenho que hidratar esse paciente no domicílio, geralmente ele vai ficar na cama. Se ele precisar ir ao banheiro ou fazer alguma coisa ele vai conseguir realizar tranquilamente com esse dispositivo. Para o domicílio, eu acho o dispositivo perfeito. (E7)*

O DHS foi considerado um facilitador no processo de transição do cuidado do paciente do setor hospitalar para os cuidados domiciliares. Além disso, também foi visualizado como uma possível estratégia para evitar a internação do paciente que está em cuidados domiciliares.

*Eu acho que ele facilita a alta do paciente que está no hospital só para hidratar. (PG2)*

*Isso que a gente fala é uma questão pontual ou, de repente, de um cuidado transicional. O paciente está internado, ele vai ter alta e você tem que treinar a família sobre como utilizar o dispositivo. Assim, você coloca uma vez e já treina, mostra à família como é que faz, como vai ser feito no domicílio. (PG6)*

*Evita a internação de quem está em casa e está desidratado. Você coloca o dispositivo e hidrata, inclusive. Você hidrata o paciente e ao tratar a desidratação, ajuda a tornar as veias mais visíveis, e o processo de cuidar do paciente, por conta desse dispositivo, é facilitado. (PG3)*

Com o objetivo de padronizar as características dos componentes do protótipo, identificaram a necessidade de definir um público-alvo inicial para o DHS. Consideraram interessante priorizar pacientes adultos, devido à menor especificidade de suas necessidades em comparação com outros grupos etários.

*Eu acho que o adulto é melhor, o pediátrico tem uma variação muito grande, tem a criança de 1 ano e a de 10, e o volume de infusão é diferente para cada um. (PG4)*

*Acho que, em um primeiro momento, o ideal seria o público adulto, porque está na fase inicial do desenvolvimento do dispositivo, criar muitas variações no momento não seria legal para o mercado. (PG5)*

O peso da bomba, aliado ao seu design arredondado e à falta de um acessório para a fixação do protótipo, foi identificado como um conjunto de fatores que contribuem para o aumento do risco de quedas do dispositivo, comprometendo, assim, a segurança do paciente.

*O que me deixa um pouco aflita é o peso da bomba, [...] qualquer movimento que possa acontecer, talvez pelo peso dela no início da infusão, pode fazer com que retraia o equipo. (E2)*

*Na hora da técnica, minha maior dificuldade foi descobrir onde colocar a bomba, porque se eu deixo aqui na cama, o paciente vai se mover na cama, vai derrubar e essa força pode romper o dispositivo. (E3)*

*Eu acho que sem o suporte, ele vai rolar e vai cair. Quando você colocou sobre a mesa, eu fiquei nervosa, porque eu penso que ele vai rolar e vai cair. (PG2)*

Para resolver essa questão e minimizar o risco de quedas do DHS, os participantes propuseram a inclusão de acessórios complementares ao kit.

*Se tivesse, também, seria um plus do dispositivo: uma bolsinha, algo assim. Para quem é o cuidador, seja cuidador profissional, ou cuidador familiar, isso ajudaria 100%, além de tentar também fazer uma exploração da independência do paciente o máximo possível.. (E2)*

*Coloca um gancho, se tiver que colocar no leito, encurta a alça, se tiver que andar, coloca uma alça maior. (PG2)*

*O ideal seria uma alça que pudesse regular o tamanho e a bolsa como um acessório que pudesse ser adquirido à parte. (PG6)*

A bomba de infusão elastomérica, inicialmente percebida como tendo um volume insuficiente, foi sugerida para ser disponibilizada em volumes variados, visando atender às diversas necessidades dos pacientes e evitar tanto o desperdício de produtos quanto de recursos financeiros.

*Se padronizarmos em um volume único, seria desprezar o soro e um sistema que custa caro de forma desnecessária; o ideal seria ter volumes diferentes para possibilitar a escolha (...) eu acho que deveria ter um produto com várias apresentações. (PG6)*

*Hoje nós temos várias bolsas de soro com volumes diferentes, por que não padronizar os volumes tradicionais? 100, 200 e 500? Se o volume prescrito for 200 e a bolsa tiver 500, haverá uma necessidade de vigiar para interromper o fluxo no período certo, seria mais um trabalho. (PG3)*

Além disso, diante da percepção de que o tempo de infusão do protótipo é longo - de 40 horas -, foi recomendado seguir os protocolos de hidratação subcutânea, como a infusão de 500 ml de SF0,9% ao longo de 24 horas.

*Eu achei o tempo de infusão muito longo [...] o dispositivo tem que ter um certo controle para que você também possa reverter a desidratação do paciente em um tempo hábil [...] eu vejo o tempo de infusão desse dispositivo como um problema. (PG3)*

*Eu acho que até 80 ml/h estará confortável, 120 não acho viável (...) normalmente, a gente infunde 500 ml em 24h, se você quiser mais volume, a gente coloca em outro sítio outra bolsa. Mas fica padronizado para 24h. (PG5)*

Todos os participantes elogiaram a agulha intravenosa selecionada para o protótipo devido à sua praticidade e segurança, apesar de tratar-se de um sítio subcutâneo. Houve consenso em padronizar o DHS com o menor calibre disponível, rejeitando a utilização de agulhas de 90º devido à dificuldade de manutenção do dispositivo no subcutâneo durante a infusão.

*Quando trabalhamos com o subcutâneo sob gravidade, o menor calibre não funciona e temos que recorrer para uns maiores. Esse dispositivo por ser por pressão, acredito que dê para diminuir o calibre, vai causar menor dor na hora da punção e vai servir para todos os pacientes. (PG6)*

*Eu acho que se fosse 90°, se não fizer uma prega adequada você pode acabar ultrapassando o tecido subcutâneo. (E2)*

*Eu não vejo problema na instalação, acredito que seria semelhante à insulina. Eu vejo problema na manutenção desse dispositivo, porque a injeção da insulina, mesmo que ele erre o local, vai ser um volume muito pequeno, é diferente de um volume de 500 ml. (PG2)*

Considerando a necessidade de simplificar o procedimento para usuários sem experiência técnica, como cuidadores informais responsáveis pela hipodermóclise no domicílio em programas de cuidados paliativos, os participantes propuseram a inclusão opcional de um guia de punção no kit do DHS. Visou-se, com isso, auxiliar, principalmente, na angulação correta de 30º ou 45º durante o processo de inserção, considerada o ponto mais crítico do procedimento.

*Acho que poderia vir até mesmo dentro do kit do dispositivo. Eu o imagino como uma peça para guiar a punção. Um lado é 15º, outro 30º e o outro 45º, e o cuidador informal vai seguir as instruções de qual grau colocar. (PG5)*

A apresentação final do protótipo possui dois *clamps* devido à junção manual dos materiais disponíveis no mercado. Embora não tenha sido intencional, essa duplicidade provocou debates entre os participantes. Após considerarem aspectos de segurança relacionados ao *clamp*, chegaram a um consenso de que a melhor opção seria posicionar o *clamp* de forma a fixá-lo próximo à bomba de infusão elastomérica e modificar sua cor para facilitar sua visualização.

*Eu daria prioridade para manter o que fica próximo da bomba, que eu acho que até exponha menos o paciente. (E6)*

*O paciente poderia até acionar o clamp sem querer, deixando-o aberto próximo do paciente ele poderia mudar de decúbito e fechar o clamp. (PG4)*

*Verdade, criar uma estratégia para tornar o clamp mais visível para os cuidadores visualizarem. (PG4)*

## Discussão

O estudo sobre o DHS evidenciou conclusões relevantes sobre o desempenho do protótipo em cenário simulado e a viabilidade dessa inovação no contexto da prática clínica. Os resultados revelaram uma recepção positiva dos profissionais de enfermagem, destacando sua facilidade de uso, segurança e potencial para otimizar a qualidade do cuidado. A satisfação com a manipulação do protótipo e a percepção de segurança associada ao sistema fechado indicam que o DHS pode oferecer uma alternativa eficaz e conveniente para a administração de fluidos em pacientes com desidratação leve a moderada. 

Ao considerar a centralidade do usuário no processo de inovação em saúde, as sugestões dos participantes para melhorar o DHS incluem: a adição de acessórios para reforçar a estabilidade do dispositivo e a disponibilização de diferentes volumes de SF0,9%, os quais poderão ser customizados futuramente de acordo com o paciente e sua necessidade, após a conclusão de estudos clínicos. Apesar da ênfase na facilidade de uso do DHS, a ausência de suporte para fixar a bomba de infusão elastomérica foi identificada como um ponto fraco, sinalizando risco potencial de complicações, como deslocamentos ou desconexões acidentais.

Apesar de a taxa de infusão atual do protótipo estar na faixa dos limites recomendados^15^, os participantes sugeriram um fluxo de 20 ml/h, considerando-o mais eficaz para o manejo da hidratação dos pacientes. Quanto ao tempo de permanência do cateter, embora seja descrito como curto pela literatura^2^, a análise de risco evidenciou que esse tempo é adequado para minimizar complicações no sítio de punção. Portanto, a padronização do DHS em 24 horas, conforme sugerido pelos participantes, aumentaria a segurança clínica e operacional do dispositivo.

Um ponto-chave identificado no estudo, que possui implicações significativas para os profissionais de saúde, foi a definição do contexto de uso e da população-alvo beneficiada pelo DHS: o ambiente domiciliar e os pacientes adultos. Os participantes visualizaram o DHS como alternativa viável para facilitar cuidados domiciliares, alinhando-se à desospitalização segura. A permanência prolongada no ambiente hospitalar aumenta os riscos para o paciente, incluindo o desenvolvimento de condições adquiridas em hospitais, destacando a desospitalização como uma meta importante para a equipe de saúde e para o próprio sistema de saúde^16^ ^-^ ^17^.

Nesse cenário, o DHS foi percebido como uma tecnologia facilitadora para a continuidade dos cuidados domiciliares, especialmente em programas onde cuidadores familiares realizam hipodermóclise sob supervisão e orientação técnica. É essencial reconhecer o papel fundamental dos cuidadores, frequentemente membros da família, que requerem suporte técnico e educação em saúde para garantir a prestação adequada de cuidados domiciliares^18^, incluindo a hidratação subcutânea. Os participantes consideram o DHS uma proposta viável para cuidadores informais, devido à sua simplicidade e características que podem facilitar o manejo seguro^19^ ^-^ ^20^.

Este estudo, exploratório e pioneiro no desenvolvimento e avaliação do DHS, revelou percepções positivas dos participantes sobre seu potencial para facilitar a desospitalização e a prevenção de hospitalizações evitáveis decorrentes da desidratação. Considerando que a hospitalização está associada à perda funcional e de capacidades locomotoras, no caso da pessoa idosa, reforça-se a relevância de estratégias que possibilitem cuidados em domicílio^21^. 

Para que esse potencial seja plenamente alcançado, será necessária a integração de conhecimentos entre as áreas da saúde e os setores industriais no processo de desenvolvimento e aprimoramento do dispositivo, como no desenvolvimento de tecnologias como o pijama hospitalar, que também contou com uma equipe interdisciplinar para sua concepção e implementação^22^.

Este estudo apresenta algumas limitações metodológicas. A amostra foi restrita a enfermeiros graduados, o que limitou a diversidade de perfis e especialidades representadas. Profissionais atuantes em pediatria, terapia intensiva e centro cirúrgico, por exemplo, não foram incluídos, o que reduziu a abrangência da avaliação quanto à aplicabilidade do DHS em diferentes cenários clínicos. Além disso, não foram incluídos usuários finais, pacientes e cuidadores, no processo de avaliação da usabilidade, o que constitui uma lacuna importante a ser superada em estudos futuros. 

Apesar dessas limitações, os resultados deste estudo oferecem subsídios relevantes para o avanço do conhecimento científico em saúde e enfermagem ao explorar a construção colaborativa de uma tecnologia voltada à administração subcutânea de fluidos para reidratação. O DHS demonstrou potencial para ampliar e fortalecer a modalidade do cuidado domiciliar, a autonomia dos pacientes e dos cuidadores e reduzir a sobrecarga nos serviços de saúde, especialmente no contexto de envelhecimento populacional e da cronicidade das doenças.

## Conclusão

A avaliação de usabilidade é crucial para identificar problemas potenciais enfrentados pelos usuários de dispositivos médicos. Este estudo demonstrou um desempenho operacional positivo do protótipo do DHS em cenário simulado, gerando uma satisfação entre os enfermeiros participantes. Embora algumas preocupações em relação ao design tenham sido destacadas, os participantes mostraram interesse na implementação do DHS em seus ambientes de trabalho. Dessa forma, os resultados deste estudo abrem caminho para futuras pesquisas envolvendo o DHS e destacam seu potencial para aprimorar os cuidados de enfermagem, especialmente entre a população adulta e idosa, e para expandir os cuidados domiciliares.

## Data Availability

Todos os dados gerados ou analisados durante este estudo estão incluídos neste artigo publicado.

# Translation: Evaluación de la usabilidad de un dispositivo innovador para hipodermoclisis en un escenario simulado con enfermeros^*^

**DOI:** 10.1590/1518-8345.8289.4929

**Authors:** Thayna de Assis Barros S. Thiago, Eduardo José Ferreira Santos, Pedro Miguel dos Santos Dinis Parreira, Anabela Salgueiro-Oliveira, Juliana Faria Campos, Inês Franco de Almeida, Rafael Alves Bernardes, Marcelle Miranda da Silva

**Affiliations:** 1 Universidade Federal do Rio de Janeiro, Escola de Enfermagem Anna Nery, Rio de Janeiro, RJ, Brasil.; 2 Bolsista da Coordenação de Aperfeiçoamento de Pessoal de Nível Superior (CAPES), Brasil.; 3 Instituto Politécnico de Viseu, Escola de Saúde, Viseu, Portugal.; 4 Escola Superior de Enfermagem de Coimbra, Unidade de Investigação em Ciências da Saúde, Enfermagem, Coimbra, Portugal.; 5 Bolsista da Escola Superior de Enfermagem de Coimbra, Unidade de Investigação em Ciências da Saúde, Enfermagem, Portugal.; 6 Universidade Católica Portuguesa, Centro de Investigação Interdisciplinar em Saúde, Lisboa, Portugal.

**Keywords:** Diseño Centrado en el Usuario, Infusiones Subcutáneas, Equipos y Suministros, Hipodermoclisis, Tecnología, Invenciones

## Abstract

**Objetivo:**

evaluar la usabilidad y el rendimiento operativo del prototipo del Dispositivo de Hidratación Subcutánea en un escenario simulado con enfermeros, así como recabar información para su refinamiento continuo.

**Método:**

estudio de usabilidad pre-experimental, cualitativo y cuantitativo, utilizando un escenario simulado, entrevista semiestructurada y grupo focal. Realizado en laboratorio, con nueve participantes con experiencia en terapia subcutánea. Se utilizó una lista de verificación como guía para llevar a cabo el escenario simulado y para la evaluación del rendimiento operativo del prototipo, así como un guion para la entrevista y el grupo focal. Se aplico el análisis de contenido en la modalidad temática.

**Resultados:**

se revelaron percepciones variadas sobre el prototipo, identificando fortalezas y debilidades. La mayoría manifestó satisfacción con la facilidad de manipulación y expresó confianza en su posible implementación en la práctica clínica. Se sugirieron mejoras relacionadas con la ergonomía y la funcionalidad, como la inclusión de accesorios para reforzar la estabilidad, además de identificar el entorno domiciliario y adultos/personas mayores como posibles contextos y beneficiarios del Dispositivo de Hidratación Subcutánea.

**Conclusión:**

el estudio evidenció un rendimiento satisfactorio del prototipo del Dispositivo de Hidratación Subcutánea en escenario simulado y generó satisfacción positiva entre los enfermeros. Se destacó el potencial del Dispositivo de Hidratación Subcutánea para mejorar la atención de enfermería, especialmente en el domicilio.

## Destacados

**(1)** El prototipo del DHS demostró eficacia en la simulación con enfermeros experimentados.

**(2)** Enfermeros manifestaron una alta satisfacción con la facilidad de uso del prototipo del DHS.

**(3)** El DHS tiene potencial para la atención domiciliaria, favoreciendo la deshospitalización.

**(4)** Se han sugerido mejoras en la ergonomía y funcionalidad para perfeccionar el DHS.

## Introducción

La deshidratación es un trastorno hídrico frecuente que afecta a los indivíduos en una variedad de escenarios, desde el ámbito domiciliário hasta el institucional^1^. Vinculada al aumento de la morbilidad y mortalidad, especialmente en grupos vulnerables como los niños y las personas mayores, destaca su relevancia y la necesidad de un enfoque cuidadoso^1^ ^-^ ^2^.

Aunque la ingesta adecuada de agua es crucial para reducir el riesgo de deshidratación, en los casos en los que la rehidratación oral no es suficiente, la administración de líquidos por vía intravenosa o subcutánea se vuelve esencial^2^. La hipodermoclisis, en particular, surge como una opción valiosa para pacientes con deshidratación leve a moderada, especialmente para aquellos con dificultades de acceso venoso o restricciones en la administración y la absorción oral^3^.

Aunque la hipodermoclisis ofrece ventajas, su aplicación sigue siendo limitada y poco utilizada debido a desafíos prácticos, especialmente en el ámbito domiciliario^2^ ^-^ ^3^. El Dispositivo de Hidratación Subcutánea (DHS) surge como una solución innovadora para mejorar la aplicación clínica de la técnica de la hipodermoclisis con seguridad y practicidad. Concebido por estudiantes de grado de una institución de enfermería de la región Centro de Portugal, como parte del módulo de innovación de la unidad curricular de Integración a la Vida Profesional, el DHS fue registrado en el Instituto Nacional de Propiedad Industrial (INPI) como modelo de utilidad, lo que destaca su relevancia para la práctica clínica contemporánea^4^.

El DHS encaja en la definición de dispositivo médico destinado a la prevención, el tratamiento o el alivio de una condición médica específica, en este contexto, la deshidratación leve a moderada, caracterizada por la pérdida de hasta un 10% de líquidos^5^. Así, siguiendo los requisitos legales establecidos por la Autoridad Nacional de Medicamentos y Productos Sanitarios (INFARMED) de Portugal y las directrices europeas, el DHS fue categorizado como un dispositivo de Clase III, clasificado específicamente como dispositivo invasivo de corta duración^6^.

En el contexto de la innovación, un prototipo es una representación física o conceptual de una idea de diseño, que pasa por etapas iterativas de prueba y perfeccionamiento para satisfacer las expectativas del usuario. Es esencial probar los prototipos para mejorar su eficacia y su usabilidad, principalmente en los dispositivos médicos^7^ ^-^ ^8^. El prototipo del DHS se desarrolló basándose en tecnologías disponibles en el mercado, siguiendo las especificaciones del modelo de utilidad del DHS (Figura 1), y fue sometido a una fase inicial de grupo focal con profesionales sanitarios para optimizar su diseño y evaluar su posible aceptación en el mercado^9^.

Según el modelo de utilidad del DHS, el dispositivo está compuesto por un sistema cerrado y prellenado con solución salina al 0,9%, listo para su uso y de un solo uso. Sin embargo, debido a la naturaleza improvisada del proceso de prototipado, se mantuvieron algunos componentes adicionales, como una pinza y el segundo lumen (4 y 9), que no se tuvieron en cuenta en la realización de este estudio.

Para el ensamblaje, se eligió una bomba de infusión elastomérica de 400 mL, con un caudal predefinido de 10 mL/h (1). El equipamiento incluye un depósito interno (2), un equipo (“juego de infusión”) (3) con pinza (4), filtro de partículas y aire (5) y dos lúmenes para la conexión (9). El dispositivo de acceso cuenta con un catéter (6), alas flexibles y ergonómicas (7) y un dispositivo de seguridad (8) para la extracción de aguja (Figura 1).

**Figura 1:** Ilustración del prototipo semifuncional del Dispositivo de Hidratación Subcutánea

Para continuar el proceso de prototipado del DHS, este estudio tiene como objetivo evaluar la usabilidad y el rendimiento operativo del prototipo del DHS en un escenario simulado con enfermeros, así como recabar información para su refinamiento continuo.

## Método

### Diseño del estudio

Se llevó a cabo un estudio preexperimental para evaluar la usabilidad del DHS, combinando enfoques cualitativos y cuantitativos, según las Directrices Metodológicas para Estudios de Evaluación de Equipamientos Médico Assistenciales^10^. Con enfoque en el ámbito operativo, analizamos la interacción usuario-dispositivo, y se desarrollaron parámetros para medir la satisfacción y el rendimiento operativo.

Realizamos un experimento de grupo único para evaluar la usabilidad del prototipo del DHS, centrado en la administración subcutánea del suero fisiológico al 0,9%, mediante una simulación clínica, seguida de entrevistas semiestructuradas. Posteriormente, los participantes fueron invitados a participar en un grupo focal con el fin de profundizar en las recomendaciones de mejora del prototipo del DHS. Para garantizar la calidad metodológica, seguimos las directrices de la extensión *Strengthening the Reporting of Observational Studies in Epidemiology* STROBE, adaptada para estudios con simulación, de acuerdo con el diseño de la investigación^11^.

### Lugar del estudio

La primera fase de este estudio tuvo lugar en el laboratorio de actividades prácticas del curso de enfermería de una universidad federal en Rio de Janeiro, Brasil. En este entorno, realizamos tanto la evaluación del prototipo en un escenario simulado como las entrevistas semiestructuradas. Por su parte, el grupo focal se llevó a cabo en el laboratorio de informática de un programa de posgrado en enfermería en la misma ciudad. Ambos laboratorios estaban reservados exclusivamente para las actividades del estudio, garantizando un entorno libre de ruido e interrupciones externas.

### Definición de la muestra

Los participantes fueron reclutados mediante la técnica *snowball*, comenzando por enfermeros que formaban parte del grupo de investigación al que estaba asociada la investigadora principal, y a partir de ellos, expandiéndose progresivamente a través de recomendaciones sucesivas de otros participantes potenciales. Este proceso se produjo en dos oleadas de recomendación, hasta que las nuevas recomendaciones comenzaron a repetir perfiles similares, sin añadir características relevantes al fenómeno estudiado^12^.

En este proceso, se indicaron 26 enfermeras, pero solo 16 eran elegibles y siete no estaban disponibles para asistir al lugar de recogida de datos. Así, la primera fase del estudio contó con nueve participantes. Aunque no existe un consenso establecido sobre el tamaño ideal de la muestra que garantice, por sí solo, una evaluación de usabilidad totalmente fiable^13^, este número se determinó por el criterio de saturación temática de los datos y por la homogeneidad de la muestra (enfermeros con experiencia en hipodermoclisis). Tras finalizar la recogida de datos, se invitó a los participantes a unirse al grupo focal. 

Durante el proceso de reclutamiento, consultamos los currículos de los enfermeros indicados y les preguntamos sobre su experiencia práctica en la técnica de hipodermoclisis. Las invitaciones se enviaron por correo electrónico y, tras su aceptación, los participantes eligieron el momento más adecuado para la recogida de datos en persona.

### Criterios de selección

La población objetivo consistió por enfermeros con experiencia en la atención a pacientes sometidos a infusiones subcutáneas, como la hipodermoclisis. Eran elegibles los enfermeros con experiencia clínica en hipodermoclisis -autodeclarada- independientemente de su área de actuación, quedando excluidos aquellos que hubieran tenido contacto previo con el producto que se iba a probar.

### Recogida de datos

Escenario simulado, entrevista semiestructurada y grupo focal

La recogida de datos se llevó a cabo entre marzo y abril de 2023, precedida por una prueba piloto realizada en septiembre de 2022, en un laboratorio de un hospital privado en Río de Janeiro. Esta prueba contó con la participación de una enfermera con experiencia en hipodermoclisis y de una docente de enfermería especializada en simulación clínica. 

Inicialmente se planificaron dos fases de prueba, pero la prueba piloto demostró que la primera - que consistía en realizar la hipodermoclisis mediante el método convencional, con una bomba de perfusión eléctrica - era innecesaria. Además, la prueba mostró la necesidad de la conexión manual entre los dispositivos para permitir la reutilización de la bomba de infusión elastomérica, ya que la unión definitiva del circuito a la aguja de un solo uso hacía inviable la reutilización. Así, la prueba en escenario simulado se centró exclusivamente en la evaluación de la usabilidad del prototipo del DHS.

El proceso de recogida de datos consistió en cuatro pasos: (1) la lectura y la firma del Formulario de Consentimiento Informado, junto con el Formulario de Consentimiento para el Uso de Imagen y Voz; (2) la caracterización del perfil profesional del participante mediante un cuestionario autoadministrado con nueve preguntas abiertas y cerradas; (3) la prueba del prototipo en un escenario clínico simulado; y (4) entrevistas semiestructuradas.

En el día y la hora programados, los participantes asistieron al laboratorio individualmente, donde recibieron una introducción sobre la investigación y el DHS. Firmaron los Formularios de Consentimiento y rellenaron el cuestionario relacionado con su perfil profesional.

En el entorno simulado, los participantes recibieron instrucciones sobre la actividad propuesta, guiadas por el guion del escenario simulado, que consistía en simular la atención a un paciente de complejidad media en una sala de clínica médica. La investigadora proporcionó un momento para que los participantes manipularan el DHS, aclararan dudas y recibieran instrucciones adicionales antes de los pasos siguientes.

Tras la introducción al escenario, los participantes simularon la aplicación de hipodermoclisis para la infusión de solución salina al 0,9% en un maniquí simulador, según las instrucciones del caso clínico. El entorno simulado fue diseñado para replicar un escenario clínico realista, incorporando recursos tecnológicos, como materiales para la hipodermoclisis y otros representativos del entorno hospitalario de una clínica médica.

El simulador seleccionado fue un maniquí bisexual, destinado a la educación en enfermería, capaz de simular una amplia gama de procedimientos básicos, incluyendo curas y vendajes, colocación de sondas, baño en cama y administración de medicamentos subcutáneos. Para los escenarios de prueba del prototipos del DHS, se utilizaron los materiales mostrados en la Figura 2.

**Figura 2:** Materiales para la prueba en el escenario simulado. Rio de Janeiro, RJ, Brasil 2023

Ítems	Cantidad
Bombas de infusión elastoméricas Easypump^®^ II LT* de B|Braun con 400 ml y flujo de infusión de 10 ml/h, con 0,9% de SF0,9% II LT*	2
Catéteres intravenosos de doble lumen, de sistema cerrado BD Nexiva™ Closed IV Catheter System	9
Rollo de apósito de película transparente impermeable Vitamedical^®^	1
Cinta adhesiva Micropore^MR^	1
Pares de guantes de procedimiento	9
Paquetes de compresas de gasa con 10 unidades cada uno	9
Botella de alcohol al 70%	1
Bolígrafos	2
Hojas con el formulario de respuestas	-
Dispositivo móvil para uso de cronómetro	1

*LT = *Long-Term*

Durante la prueba, los participantes fueron observados por la investigadora principal, que utilizó una lista de verificación basada en el Procedimiento Operativo Estándar (POE) de un hospital universitario de Río de Janeiro para la hipodermoclisis y que fue sometida a una prueba piloto para asegurar la alineación con la práctica de enfermería. Esta lista de verificación verificó los pasos obligatorios de la instalación subcutánea con el DHS: lavado de manos, uso de guantes, antisepsia cutánea, formación del pliegue, inserción del catéter, apertura de las pinzas y fijación del dispositivo con apósito de película estéril.

La tarea se consideró concluida con éxito cuando se completaban todos los pasos obligatorios y el participante declaró verbalmente la finalización del procedimiento. Además de registrar errores/éxitos y el tiempo dedicado a la simulación, la lista de verificación evaluó el rendimiento operativo del prototipo, tal como se detalla en la sección de resultados (Tabla 2).

Tras completar la prueba, los participantes realizaron una entrevista semiestructurada para evaluar su satisfacción con el uso del prototipo del DHS y compartir percepciones y contribuciones adicionales. Así, la satisfacción se midió en función de las impresiones positivas y negativas expresadas durante las entrevistas, que se grabaron en audio y se transcribieron íntegramente, poniendo fin al proceso de recogida de datos en esta reunión.

El grupo focal tuvo lugar el 19 de abril de 2023, con la investigadora principal como moderadora, una estudiante de máster como observadora y seis participantes previamente entrevistados. De los nueve invitados, tres declinaron la invitación por incompatibilidad de agenda, sin perjuicio a la diversidad temática, ya que los ejes tratados ya habían surgido en las entrevistas individuales.

Para la conducción del grupo focal, se elaboró un guion semiestructurado y detallado, que presentaba los pasos a seguir. Inicialmente, los participantes se reunieron y la investigadora introdujo la propuesta y el formato de la sesión. Durante este momento inicial, los participantes tuvieron la oportunidad de presentarse e interactuar antes del inicio efetivo de la actividad.

Después, la investigadora dirigió la discusión según las preguntas previstas, asegurando la participación de todos y abriendo espacio para preguntas adicionales. Las preguntas abordaron cuatro ejes temáticos: características generales del DHS; componentes del DHS; contexto de uso del DHS y la gestión y gerencia de enfermería. El grupo focal duró una hora y veinte minutos, terminando cuando se agotaron todas las preguntas. El guion completo está disponible como material complementario.

### Tratamiento y análisis de datos

Los audios de las entrevistas semiestructuradas y del grupo focal fueron transcritos íntegramente y analizados conjuntamente, ya que presentaban contenidos complementarios. Los datos cuantitativos de los cuestionarios y de la lista de verificación se analizaron utilizando el *software Statistical Program for the Social Sciences* (SPSS), versión 22.0, mediante análisis descriptivo.

Las entrevistas y el grupo focal fueron transcritos íntegramente por la investigadora principal y enviados a los participantes para su validación, sin que se solicitaran cambios por su parte. El análisis de datos cualitativos se realizó utilizando el *Qualitative Data Analysis Software* (webQDA^®^) por el grupo de investigadores, siguiendo el método de análisis de contenido temático propuesto por Bardin: preanálisis, exploración del material y tratamiento de los resultados obtenidos, inferencia e interpretación^14^. Los resultados se organizaron en unidades temáticas definidas a priori, basadas en los objetivos del estudio, a saber: satisfacción, seguridad, contexto de la aplicación/público objetivo y componentes del prototipo.

### Consideraciones éticas

El proyecto recibió la aprobación por el Comité de Ética en la Investigación de la Escuela de Enfermería Anna Nery/Instituto de Atención de la Salud de São Francisco de Assis/Universidad Federal de Río de Janeiro (CEP-EEAN/HESFA/UFRJ) (Dictamen n.º 5.786.249), cuya dictamen se adjunta a esta presente sumisión. Todos los participantes se mantuvieron en el anonimato, identificados mediante códigos específicos: “E” para enfermeros, seguido de un número, en las entrevistas semiestructuradas; y “PG”, para los participantes del grupo focal, seguido de un número. Esta medida se tomó para preservar la integridad y la privacidad de los participantes.

## Resultados

En cuanto al perfil profesional de los nueve participantes, expuesto en la Tabla 1, la mayoría (66,7%) eran mujeres, con una edad media de 33 años, que oscilaba entre 27 y 64 años. Cinco tenían un máster (55,6%) y uno, doctorado (11,1%). La mayoría (66,7%) tenía más de seis años de experiencia como enfermeros. Cinco tenían entre uno y cinco años de experiencia en hipodermoclisis, y solo uno tenía más de diez años. Las áreas de experiencia incluían oncología (la más común, con cinco participantes), cuidados paliativos, urgencias y gerontología.

La actividad de simulación, observada con la ayuda de la lista de verificación (Tabla 2) y cronometrada, reveló un tiempo medio de ejecución por parte de los participantes de 5 minutos y 42 segundos. El tiempo máximo registrado para la conclusión fue de 10 minutos y 2 segundos, mientras que el mínimo fue de 3 minutos y 24 segundos, conforme ilustrado en la Figura 3. Todos los participantes completaron la simulación con éxito, aunque dos no verificaron si las pinzas estaban cerradas.

**Tabla 1:** Datos sociodemográficos y profesionales de los participantes. Rio de Janeiro, RJ, Brasil, 2023

Variables	Frecuencia (n)	Porcentaje (%)
Edad (años)		
27-44	6	66.7
45-64	3	33.3

Sexo		
Femenino	6	66.7
Masculino	3	33.3

Máximo grado académico		
Especialización	3	33.3
Máster	5	55.6
Doctorado	1	11.1

Tiempo ejerciendo el cargo de enfermero		
2 to 5 años	3	33.3
6 to 10 años	2	22.2
Más de 10 años	4	44.4

Cuánto tiempo de experiencia tiene con hipodermoclisis		
Menos de 1 año	2	22.2
1 to 5 años	5	55.6
6 to 10 años	1	11.1
Más de 10 años	1	11.1

Ha recibido alguna formación en hipodermoclisis		
Sí	8	88.9
No	1	11.1

¿Qué nivel de conocimiento considera tener en relación a la hipodermoclisis?		
Excelente	1	11.1
Muy bueno	2	22.2
Bueno	6	66.7

**Tabla 2:** Lista de verificación de los procedimientos realizados en la fase de prueba en escenario simulado. Rio de Janeiro, RJ, Brasil 2023

Procedimientos	Frecuencia (n)	Porcentaje (%)
Lavarse las manos con agua y jabón o aplicar alcohol al 70% en las manos	9	100
Ponerse los guantes de procedimiento	9	100
Abrir el paquete del prototipo del Dispositivo de Hidratación Subcutánea	9	100
Asegurarse de que las pinzas estén cerradas		
Sí	7	77.8
No	2	22.2
Realizar antisepsia de la piel con gasa remojada en clorhexidina acuosa al 2%	9	100
Traccionar un pliegue de piel para acceso subcutáneo	9	100
Introducir el catéter sin aguja en un ángulo de 30º a 45º con el bisel hacia arriba	9	100
Abrir las pinzas de la línea de extensión y liberar el flujo	9	100
Fijar el dispositivo con película transparente estéril	9	100
Completó la tarea	9	100

**Figura 3:** Tiempo de ejecución del procedimiento de prueba los enfermeros en la simulación

Los participantes expresaron satisfacción por la facilidad de manejo del dispositivo, señalando que, incluso sin recibir formación previa sobre su funcionamiento, no encontraron dificultades durante la simulación.

*Me pareció genial, compacta y bonita. Creo que es muy fácil de manejar, muy buena, y no tengo ninguna dificultad para usarla. (E5)*

*Me pareció muy interesante y, sobre todo, fácil de usar, intuitivo y, sobre todo, no te da margen para que lo estés manipulando y, sobre todo, para cambiar ningún objetivo prefabricado o previo a su uso. (E8)*

La innovación que incorpora la bomba de infusión elastomérica se consideró la característica central del prototipo del DHS. Además de contribuir a la composición de un sistema compacto y simplificar la preparación/uso de materiales, esta innovación sustituye a equipos complejos y voluminosos, como la bomba de perfusión eléctrica.

*Ciertamente, el primer punto es que la bomba de infusión es una obra que tienes que desplazar y es difícil de manejar, así como el circuito gravitatorio, que muchos no entienden cómo funciona y acaban bajándolo, y esto no es efectivo. Esta bomba es más fácil, solo hay que ponerla y ya está. (E2)*

*Algo que considero que es súper importante: eliminamos el uso de la bomba de infusión, que es una tecnología dura y cara, difícil de usar y de manejar, además de ser difícil de transportar y que hace un ruido insoportable y molesta al paciente. (PG3)*

Cuando se les preguntó sobre la intención de uso, los participantes expresaron confianza en que el prototipo del DHS contribuiría significativamente para sus actividades profesionales cuando se integrara en el entorno laboral, evidenciando su satisfacción y entusiasmo ante la perspectiva de su implementación.

*Así que, para mí, estaría encantado de usarlo, sin mencionar lo se dejar el dispositivo instalado y todo eso, para el paciente estable; es genial. (E5)*

*Creo que va a ser muy útil, sí. (E6)*

*¡Es perfecto! ¿Para el hogar? Perfecto. ¿Para el cuidador domiciliario? Creo que es perfecto. (E7)*

El prototipo recibió elogios unánimes de todos los participantes. Se destacó su capacidad para garantizar un entorno libre de contaminación, gracias a su diseño como sistema cerrado, así como a su facilidad de manejo, tanto para profesionales como para cuidadores.

*Creo que el nivel de infección es muy bajo, si yo fuera jefe de planta, estaría muy feliz [...] Me pareció interesante no haber tenido contacto directo con la aguja. Si es por una infección, probablemente será por la persona que realizó la técnica [...] por lo que sería menos problemático para el enfermería de calidad. (E5)*

*En cuanto al proceso, creo que acaba siendo el mismo proceso que el resto del equipo. Pero en relación con el paciente, porque es un sistema cerrado, de no tener que manipular, y de no cambiar el tema del goteo o del tiempo de infusión [...]. Creo que es más seguro para el paciente. (E9)*

*Lo que favorece la seguridad del dispositivo es, por ejemplo, que es un sistema cerrado y no se puede cambiar el mL/H. En casa, pueden abrirse sin querer y generar ese edema en el subcutáneo. La seguridad es: independientemente de la altura del dispositivo, funcionará, porque funciona bajo presión, y independientemente del movimiento que se realice, el ml/h estará garantizado. (PG6)*

La propuesta del DHS como dispositivo listo para usar fue reconocida como una ventaja significativa, puesto que simplifica las tareas de los profesionales de salud y favorece la seguridad del paciente.

*Creo que es muy positivo e interesante, especialmente porque no controla el ml/h y viene prefabricado. Ya estamos seguros de lo que se infundirá. (E8)*

*Creo que facilita el trabajo del equipo, llega completo y es más rápido. (PG2)*

Para mantener este sistema inviolable y mitigar las posibilidades de mal uso del dispositivo, los participantes identificaron debilidades en el prototipo que podrían modificarse para garantizar la seguridad propuesta.

*Me pareció que el puerto de entrada de la bomba elastomérica era muy fácil de abrir. Esto va a pasar por otras manos [...] existe el riesgo de contaminar el sistema durante el llenado y de romper la esterilidad del dispositivo; si esto continúa así, hay que dejarlo cerrado. (PG3)*

*Habrá gente en el domicilio que tomará una jeringuilla e intentará inyectar algo aquí. (PG5)*

*Creo que la parte superior de la bomba podría seguir el mismo patrón que la inferior. Debe haber aquí la parte donde se pone el suero y luego pones una tapa cerrada, para que nadie ponga nada. (PG4).*

Los participantes del estudio mostraron interés en implementar el DHS en sus lugares de trabajo. Más concretamente, los profesionales que trabajan en la prestación de servicios de atención domiciliaria, que abarcan tanto cuidados paliativos oncológicos como geriátricos, destacaron la posible relevancia del DHS en este contexto. Enfatizaron, sobre todo, que el dispositivo sería bien aceptado para la atención domiciliaria, en la que los pacientes reciben atención de familiares o cuidadores informales.

*Creo que será un dispositivo muy beneficioso, especialmente para uso domiciliario y en atención privada, o quizá incluso para una institución de cuidados a largo plazo [...]. Ahora, hablando desde el punto de vista de la gerontología, para evitar la hospitalización, a veces solo quieres ver si el problema de ese paciente es la deshidratación [...] Creo que es perfecto. (E6)*

*En el domicilio, creo que es perfecto, por la seguridad, por la practicidad del manejo, incluso por el aspecto estético es agradable. En casa no tienen soporte sérico, se adaptan constantemente para hacerlo por gravedad. [...] Así que, si tengo que hidratar a este paciente en domicilio, normalmente se queda en la cama. Si necesita ir al baño o hacer algo, podrá moverse con tranquilidad con este dispositivo. Para el domicilio, creo que el dispositivo es perfecto. (E7)*

El DHS fue considerado un facilitador en el proceso de transición de la atención del paciente del sector hospitalario al cuidado domiciliario. Además, también se consideró una posible estrategia para evitar la hospitalización de pacientes que están en cuidados domiciliarios.

*Creo que facilita el alta del paciente que está en el hospital solo para recibir hidratación. (PG2)*

*De lo que hablamos es de un problema puntual o, de vez en cuando, de cuidados de transición. El paciente es hospitaliza, se le dará el alta y hay que formar a la familia sobre cómo usar el dispositivo. Así que te lo pones una vez y entrenas, enseñas a la familia cómo hacerlo, cómo se hará en casa. (PG6)*

*Evita la hospitalización de quienes están en casa y deshidratados. Incluso te pones el dispositivo y te hidratas. Hidratas al paciente y, al tratar la deshidratación, ayudas a que las venas sean más visibles, facilitando así el proceso de cuidado del paciente, gracias a este dispositivo. (PG3)*

Con el objetivo de estandarizar las características de los componentes prototipo, identificaron la necesidad de definir un público objetivo inicial para el DHS. Consideraban interesante priorizar a los pacientes adultos, debido a la menor especificidad de sus necesidades en comparación con otros grupos de edad.

*Creo que el adulto es mejor, el pediátrico tiene una gran variación, hay niños de 1 año y de 10 años, y el volumen de infusión varía para cada uno. (PG4)*

*Creo que, al principio, lo ideal sería el público adulto, porque está en la fase inicial del desarrollo del dispositivo, crear muchas variaciones en este momento no sería lo más conveniente para el mercado. (PG5)*

El peso de la bomba, unido a su diseño redondeado y la ausencia de un accesorio para fijar el prototipo, se identificó como un conjunto de factores que contribuyen al aumento del riesgo de caídas del dispositivo, comprometiendo así la seguridad del paciente.

*Lo que me pone un poco nerviosa es el peso de la bomba, [...] cualquier movimiento que pueda ocurrir, quizá debido a su peso al principio de la infusión, puede hacer que el equipo se retraiga. (E2)*

*En el momento de la técnica, mi mayor dificultad fue encontrar dónde colocar la bomba, porque si la dejo aquí en la cama, el paciente se mueve sobre la cama, la derriba y esta fuerza puede romper el dispositivo. (E3)*

*Creo que, sin el soporte, rodará y caerá. Cuando lo pones sobre la mesa, estaba nerviosa, porque creo que va a rodar y caer. (PG2)*

Para resolver esa cuestión y minimizar el riesgo de caídas por DHS, los participantes propusieron la inclusión de accesorios complementarios al kit.

*Si también lo hiciera, sería un punto a favor del dispositivo: una pequeña bolsa, algo así. Para quien sea el cuidador, ya sea un cuidador profesional o un cuidador familiar, esto ayudaría al 100%, además de intentar explorar la independencia del paciente tanto como sea posible. (E2)*

*Pon un gancho, si tienes que ponerlo en la cama, acorta el mango; si tienes que andar, pon un mango más largo. (PG2)*

*Lo ideal sería una correa que pudiera regular el tamaño y una bolsa como accesorio que pudiera comprarse por separado. (PG6)*

Se sugirió que la bomba de infusión elastomérica, inicialmente percibida como de volumen insuficiente, se sugirió estar disponible en volúmenes variables, con el fin de satisfacer las diversas necesidades de los pacientes y evitar tanto el desperdicio de productos como el de recursos financieros.

*Si lo estandarizamos en un solo volumen, sería un suero despreciable y un sistema que cuesta innecesariamente; Lo ideal sería tener diferentes volúmenes para que la elección fuera posible (...) creo que debería haber un producto con varias presentaciones. (PG6)*

*Hoy en día disponemos varias bolsas de suero con diferentes volúmenes, ¿por qué no estandarizar los volúmenes tradicionales? ¿100, 200 y 500? Si el volumen prescrito es 200 y la bolsa tiene 500, habrá que vigilar para detener el flujo en el momento adecuado, sería un trabajo adicional. (PG3)*

Además, dada la percepción de que el tiempo de infusión del prototipo es largo - 40 horas - se recomendó seguir protocolos de hidratación subcutánea, como la infusión de 500 ml solución salina al 0,9%, durante 24 horas.

*Me pareció que el tiempo de infusión era demasiado largo [...] el dispositivo debe tener cierto control para que también se puedas revertir la deshidratación del paciente en un tiempo hábil [...] Veo el tiempo de infusión de este dispositivo como un problema. (PG3)*

*Creo que hasta 80 ml/h será cómodo, 120 no creo que sea factible (...) normalmente, infundimos 500 ml en 24 horas, si quieres más volumen, ponemos otra bolsa en otro sitio. Pero está estandarizado a 24 horas. (PG5)*

Todos los participantes elogiaron la aguja intravenosa seleccionada para el prototipo por su practicidad y seguridad, a pesar de que se tratara de un sitio subcutáneo. Se consiguió estandarizar el DHS con el calibre más pequeño disponible, rechazando el uso de agujas de 90º debido a la dificultad para mantener el dispositivo subcutáneamente durante la infusión.

*Cuando trabajamos con el subcutáneo bajo gravedad, el calibre más pequeño no funciona y tenemos que recurrir a los más grandes. Este dispositivo, por ser de presión, creo que se puede reducir el calibre, causará menos dolor en el momento de la punción y servirá a todos los pacientes. (PG6)*

*Creo que si estaba a 90°, si no haces un pliegue adecuado puedes acabar yendo más allá del tejido subcutáneo. (E2)*

*No veo problema en la instalación, creo que sería similar a la insulina. Veo un problema en el mantenimiento de este dispositivo, porque la inyección de insulina, aunque no encuentre la localización, será de un volumen muy pequeño, es diferente de un volumen de 500 ml. (PG2)*

Teniendo en cuenta la necesidad de simplificar el procedimiento para usuarios sin experiencia técnica, como los cuidadores informales responsables de la hipodermoclisis en casa en programas de cuidados paliativos, los participantes propusieron la inclusión opcional de una guía de punción en el kit del DHS. Con ello pretendió, sobre tudo, ayudar en el ángulo correcto de 30º o 45º durante el proceso de inserción, que se considera el punto más crítico del procedimiento.

*Creo que incluso podría venir dentro del kit del dispositivo. Me lo imagino como una pieza para guiar la punción. Un lado es 15º, otro de 30º y el otro de 45º, y el cuidador informal seguirá las instrucciones sobre qué grado colocar. (PG5)*

La presentación final del prototipo tiene dos pinzas debido a la unión manual de los materiales disponibles en el mercado. Aunque no fue intencionado, esta duplicidad generó debates entre los participantes. Tras considerar los aspectos de seguridad relacionados con la pinza, llegaron a un consenso en que la mejor opción sería colocarla de forma que se fijara cerca de la bomba de infusión elastomérica y modificar su color para facilitar su visualización.

*Yo daría prioridad a mantener lo que está cerca de la bomba, que creo que incluso expone menos al paciente. (E6)*

*El paciente incluso podría activar la pinza sin querer, dejándola abierta cerca de él y podía cambiar el decúbito y cerrar la pinza. (PG4)*

*Cierto, crea una estrategia para hacer la pinza más visible para que los cuidadores puedan visualizarla (PG4)*

## Discusión

El estudio sobre el DHS evidenció conclusiones relevantes sobre el rendimiento del prototipo en un escenario simulado y la viabilidad de esta innovación en el contexto de la práctica clínica. Los resultados revelaron una acogida positiva por parte de los profesionales de enfermería, destacando su facilidad de uso, seguridad y potencial para optimizar la calidad de la atención. La satisfacción con la manipulación del prototipo y la percepción de seguridad asociada al sistema cerrado indican que el DHS puede ofrecer una alternativa eficaz y conveniente para la administración de líquidos en pacientes con deshidratación leve a moderada.

Teniendo en cuenta la centralidad del usuario en el proceso de innovación en salud, las sugerencias de los participantes para mejorar el DHS incluyen: la adición de accesorios para reforzar la estabilidad del dispositivo y la disponibilidad de diferentes volúmenes de solución salina al 0,9%, los cuales podrán ser personalizados futuramente de acuerdo con el paciente y sus necesidades, tras la conclusión de los estudios clínicos. A pesar del énfasis en la facilidad de uso del DHS, la ausencia de soporte para fijar la bomba de infusión elastomérica fue identificada como un punto débil, señalando un posible riesgo de complicaciones como desplazamientos o desconexiones accidentales.

Aunque la tasa actual de infusión del prototipo se encuentra dentro del rango de los límites recomendados^15^, los participantes sugirieron un caudal de 20 ml/h, que se consideró más eficaz para el manejo de la hidratación de los pacientes. En cuanto al tiempo de permanencia del catéter, aunque en la literatura se describe como corto^2^, el análisis de riesgo demostró que este tiempo es suficiente para minimizar complicaciones en el lugar de la punción. Por lo tanto, estandarizar el DHS a las 24 horas, como sugirieron los participantes, aumentaría la seguridad clínica y operativa del dispositivo.

Un punto clave identificado en el estudio, que tiene implicaciones significativas para los profesionales de la salud, fue la definición del contexto de uso y de la población objetivo beneficiada por el DHS: el entorno domiciliario y los pacientes adultos. Los participantes consideraron el DHS como una alternativa viable para facilitar la atención domiciliaria, alineándose con una deshospitalización segura. La estancia prolongada en el entorno hospitalario aumenta los riesgos para el paciente, incluyendo el desarrollo de condiciones adquiridas en hospitales, destacando la deshospitalización como un objetivo importante para el equipo de salud y para el propio sistema de salud^16^ ^-^ ^17^.

En este escenario, el DHS se percibió como una tecnología facilitadora para la continuidad de la atención domiciliaria, especialmente en programas donde los cuidadores familiares realizan hipodermoclisis bajo supervisión y orientación técnica. Es fundamental reconocer el papel fundamental de los cuidadores, a menudo familiares, que requieren apoyo técnico y educación en salud para garantizar la provisión adecuada de cuidados domiciliarios^18^, incluida la hidratación subcutánea. Los participantes consideran que el DHS es una propuesta viable para cuidadores informales, debido a su simplicidad y características que pueden facilitar un manejo seguro^19^ ^-^ ^20^.

Este estudio exploratorio, pionero en el desarrollo y evaluación del DHS, reveló percepciones positivas de los participantes sobre su potencial para facilitar la deshospitalización y la prevención de hospitalizaciones evitables debidas a la deshidratación. Teniendo en cuenta que la hospitalización se asocia con la pérdida de función y capacidades locomotoras, en el caso de las personas mayores, se refuerza la relevancia de las estrategias que permiten el atención domiciliaria^21^.

Para que este potencial se alcance plenamente, será necesario integrar el conocimiento entre las áreas de salud y los sectores industriales en el proceso de desarrollo y mejora del dispositivo, así como en el desarrollo de tecnologías como el pijama hospitalario, que también contó con un equipo interdisciplinar para su concepción e implementación^22^.

Este estudio presenta algunas limitaciones metodológicas. La muestra se restringió a enfermeros graduados, lo que limitó la diversidad de perfiles y especialidades representados. Por ejemplo, no se incluyeron profesionales que trabajaban en pediatría, cuidados intensivos y quirófano, lo que redujo el alcance de la evaluación sobre la aplicabilidad del DHS en diferentes escenarios clínicos. Además, los usuarios finales, pacientes y cuidadores no fueron incluidos en el proceso de evaluación de usabilidad, lo que constituye una laguna importante a superar en futuros estudios.

A pesar de estas limitaciones, los resultados de este estudio ofrecen aportes relevantes para el avance del conocimiento científico en salud y enfermería al explorar la construcción colaborativa de una tecnología orientada a la administración subcutánea de líquidos para la rehidratación. El DHS ha demostrado potencial para ampliar y fortalecer la modalidad de atención domiciliaria, la autonomía de los pacientes y de los cuidadores, y reducir la carga sobre los servicios de salud, especialmente en el contexto del envejecimiento poblacional y las enfermedades crónicas.

## Conclusión

La evaluación de usabilidad es crucial para identificar posibles problemas que enfrentan los usuarios de dispositivos médicos. Este estudio demostró un rendimiento operativo positivo del prototipo del DHS en un escenario simulado, suscitando satisfacción entre los enfermeros participantes. Aunque se destacaron algunas preocupaciones de diseño, los participantes mostraron interés en implementar el DHS en sus entornos de trabajo. Así, los resultados de este estudio abren camino para futuras investigaciones relacionadas con el DHS y destacan su potencial para mejorar la atención de enfermería, especialmente en la población adulta y mayor, y para ampliar la atención domiciliaria.

## Data Availability

Todos los datos generados o analizados durante este estudio están incluidos en este artículo publicado.
